# Bacteriophage Therapy for *Aeromonas salmonicida*: A Systematic Evaluation of Translational Readiness in Aquaculture

**DOI:** 10.3390/microorganisms14102274

**Published:** 2026-10-06

**Authors:** Soni Andriawan, Karin Schwaiger, Astrid S. Holzer, Mona Saleh

**Affiliations:** 1 Division of Fish Health, Department of Farm Animals and Veterinary Public Health, University of Veterinary Medicine, 1210 Vienna, Austria; soni.andriawan@vetmeduni.ac.at (S.A.);; 2 Department of Aquaculture, University of Muhammadiyah Malang, Malang 65144, Indonesia; 3 Unit of Food Hygiene and Technology, Department of Farm Animals and Veterinary Public Health, University of Veterinary Medicine Vienna, 1210 Vienna, Austria

**Keywords:** antimicrobial resistance (AMR), aquaculture disease management, bacterial resistance mechanisms, furunculosis, lytic infection cycle, phage-host interaction

## Abstract

*Aeromonas salmonicida* is an important threat to the sustainability of aquaculture production and a major contributor to antimicrobial resistance, which has encouraged bacteriophage therapy. This review evaluated the current evidence base for bacteriophage therapy against *A*. *salmonicida*. Literature searches were carried out in Scopus, PubMed, and Web of Science prior to 2 April 2026, according to PRISMA 2020 guidelines. After removing 46 duplicate records, a final dataset of 108 unique publications was obtained for analysis. A data extraction form was created to include studies examining bacteriophage activity against *A. salmonicida*. Information about phage properties‚ experimental conditions‚ treatment outcomes, and study methods was extracted. Study quality and translation potential were evaluated with a qualitative appraisal tool, and a narrative synthesis was chosen due to significant heterogeneity. A total of 22 studies met the inclusion criteria; however, most were conducted in vitro, with few in vivo. Because of heterogeneity in phage–host systems, study designs, and outcomes, systems cannot be compared. Overall‚ bacteriophage therapy is a promising targeted treatment to address *A*. *salmonicida* infection. Evidence from these studies suggests more controlled studies‚ phage genome safety assessment‚ evaluation of phage resistance development‚ and field trials are necessary before the adoption of these treatments.

## 1. Introduction: The Threat of *Aeromonas salmonicida* to Aquaculture

Aquaculture is one of the fastest-growing sectors in global food production. It plays an important role in meeting the demand for animal protein [1,2]. However, the intensification of aquaculture industries has led to a drawback: the spread of infectious diseases, which has inhibited the sustainability of fish production [3]. In those case, *Aeromonas* spp. are responsible for global economic losses through excess mortality and increased expenditure for disease prevention and treatment [4,5,6,7,8]. As an example, *Aeromonas salmonicida*‚ the causative agent of furunculosis‚ is one of the most influential bacterial fish pathogens infecting many fish species‚ including salmonids [9,10,11,12].

Treatment and control of *A*. *salmonicida* infections historically involved the use of antibiotics‚ but the uncontrolled and frequent use of antibiotics in aquaculture has led to the emergence of antimicrobial resistance (AMR) in *A*. *salmonicida*‚ which is a direct threat to aquatic health and poses a public health risk [13,14]. Concerns about antibiotic residues in water systems and fish products‚ increased regulatory restrictions‚ and higher consumer demand for environmentally friendly technologies have made research on new antimicrobial technologies a high priority among many areas of research in both the public and private sectors [15,16,17].

Bacteriophage (phage) therapy‚ while still experimental‚ is emerging as a possible alternative to antibiotics due to its specificity to bacteria‚ ability to self-replicate‚ and ability to target antibiotic-resistant bacteria [18,19]. The therapy employs viruses that infect and lyse bacteria‚ and is thus a biologically targeted approach to the treatment of infections [20,21]. In aquaculture‚ the therapy has been used predominantly against *Vibrio* [22], *Pseudomonas* [23] and *Aeromonas* [24], with variable success in vitro and in experimental fish. Bacteriophages against *A*. *salmonicida* have been found to cause a high level of bacterial lysis in vitro and‚ in some studies‚ to improve the survival of infected fish [25,26].

Despite these advances‚ phage therapy translational progress from pipeline experiments to industrial application in aquaculture is incomplete‚ and most reported experiments are carried out in vitro [27,28]. Very few experiments have investigated the in vivo efficacy of phages under aquaculture environment conditions. Key factors that influence the deployability of phages in aquaculture, such as host range, environmental stability, delivery methods, regulatory requirements, and others, are often reported inconsistently or not at all. This inconsistency makes it challenging to accurately assess their real-world efficacy [29,30,31].

Meeting this demand requires a thorough evaluation of the existing literature and a series of experimental trials. Although several reviews have provided an overview of the efficacy of phage therapy in aquaculture‚ few have quantitatively assessed translational readiness across multiple axes. In particular‚ there is a lack of mechanistic models linking biological efficacy and practical aspects of aquaculture practice.

Therefore, the present study addressed this gap by performing a bibliometric and systematic literature review (SLR) of bacteriophage therapy against *A. salmonicida* and by developing a Phage Therapy Readiness Framework (PTRF) as an exploratory, semi-quantitative approach for mapping translational evidence across five domains: efficacy, host coverage and resistance robustness, environmental stability, delivery and formulation, and translational feasibility. This framework is intended to organize heterogeneous evidence and identify translational gaps rather than serve as a validated predictor of commercial, regulatory, or field readiness.

## 2. Materials and Methods

### 2.1. Study Design and Data Sources

This study employed an integrated analytical approach, combining bibliometric analysis and an SLR, to evaluate global trends and experimental evidence on bacteriophage therapy against *A. salmonicida*. In addition, this study also developed and applied the PTRF to assess the translational maturity of the identified studies.

The literature search was performed across three major scientific databases, including Web of Science (WoS), Scopus, and PubMed, using the Boolean query (“*Aeromonas salmonicida*” OR “*A. salmonicida*”) AND (phage OR bacteriophage OR “phage therapy” OR “phage treatment” OR “phage-based”), which was executed on 2 April 2026. The search was restricted to English-language publications between 1 January 2015 and 31 December 2025, and to peer-reviewed journal articles to ensure scientific quality and relevance. Publications from 2026 were intentionally excluded because the search was performed early in the calendar year and therefore represented only a partial publication year. The present study restricted the search to complete calendar years to maintain temporal consistency, particularly for the bibliometric analysis of annual publication trends.

The review was not prospectively registered, and no formal review protocol was prepared before the search. This limitation is acknowledged in the interpretation of the findings. The review process nevertheless followed a predefined search strategy and eligibility criteria, and reporting followed PRISMA 2020.

### 2.2. Data Collection and Processing

A total of 154 records were initially identified across the three databases (PubMed: 26; Scopus: 50; WoS: 78). The retrieved datasets were exported and processed utilizing the Bibliometrix package in R (version 2026.01.1, Build 403). Afterward, the databases were merged, and duplicate records were removed to ensure accuracy and avoid redundancy. After removing 46 duplicate records, the final dataset comprised 108 unique publications for analysis. These publications formed the bibliometric dataset and were subsequently subjected to the systematic-review screening procedure.

The bibliometric dataset (*n* = 108) includes studies retrieved via keyword matching, some of which may not directly focus on *A. salmonicida* or experimental phage applications. The present study retained these records to ensure a comprehensive representation of the research landscape. In contrast, the systematic literature review applied additional inclusion criteria, retaining only peer-reviewed studies that specifically investigated bacteriophage targeting *A. salmonicida* and reported primary experimental data (in vitro and/or in vivo), resulting in a final subset of 22 studies.

S.A. primarily conducted study screening, data extraction, assessment of methodological quality, and PTRF scoring. The authors discussed these steps with K.S. and M.S. during methodological and analytical reviews. K.S. provided methodological oversight, while M.S. handled formal analysis and overall analytical guidance. A.S.H. contributed to manuscript review and editing. The authors collaboratively addressed uncertainties related to study eligibility, methodological evaluation, and PTRF scoring through these discussions. Data extraction followed predefined variables, and methodological assessment and PTRF scoring followed the criteria in Supplementary Tables S2 and S3.

### 2.3. Bibliometric Analysis

The retrieved data were exported to compatible formats and then analyzed using the Bibliometrix package (R version 2026.01.1) and the Biblioshiny interface. The bibliometric analysis focused on identifying annual publication trends, leading countries and research institutions, collaboration networks, and keyword co-occurrence patterns. This approach enables a comprehensive mapping of the intellectual structure and emerging trends in phage therapy research targeting *A. salmonicida*.

### 2.4. Systematic Literature Review

A systematic literature review (SLR) was conducted in accordance with the Preferred Reporting Items for Systematic Reviews and Meta-Analyses (PRISMA 2020) guidelines to evaluate experimental evidence on bacteriophage therapy targeting *A. salmonicida* [32]. The review was not prospectively registered, and a review protocol was not prepared. This analysis used the same dataset of 108 publications for a multi-stage screening process.

**Figure 1:**
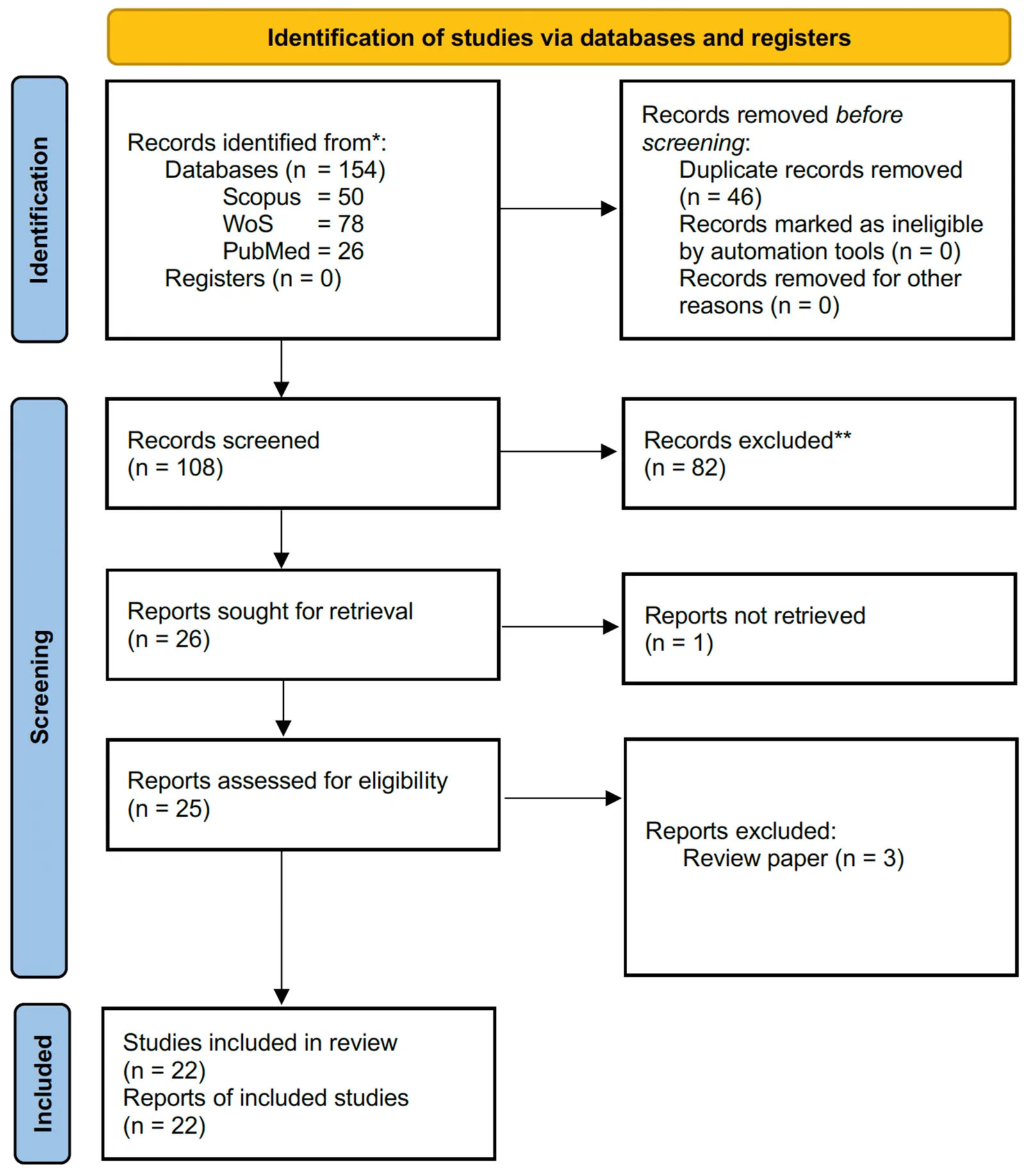
PRISMA flow diagram illustrating the study (2015–2025) selection process for bacteriophage research targeting *A. salmonicida*. * Raw records identified from the three databases (Scopus, Web of Science, and PubMed). ** Records remaining after removal of duplicates and abstract screening.

The 108 unique records were subjected to title and abstract screening. Eighty-two records [24,30,33,34,35,36,37,38,39,40,41,42,43,44,45,46,47,48,49,50,51,52,53,54,55,56,57,58,59,60,61,62,63,64,65,66,67,68,69,70,71,72,73,74,75,76,77,78,79,80,81,82,83,84,85,86,87,88,89,90,91,92,93,94,95,96,97,98,99,100,101,102,103,104,105,106,107,108,109,110,111,112] were excluded because they did not specifically address bacteriophage applications involving *A. salmonicida*. The process then evaluated 26 reports; one could not be retrieved [113], and three were review articles [114,115,116] and were therefore excluded (Table S1). Consequently, 22 primary experimental studies met the eligibility criteria and were included in the qualitative synthesis (Table 1). The complete study selection process is shown in the PRISMA flow diagram in Figure 1.

#### 2.4.1. Inclusion and Exclusion Criteria

Studies that investigated bacteriophages targeting *A. salmonicida*, reported primary experimental data (including in vitro and in vivo), were published in peer-reviewed journals, and fell within the defined time frame (2015–2025) were included. These criteria ensured that only relevant, high-quality studies that directly contributed to the understanding of phage therapy against *A. salmonicida* were considered.

This study also excluded review articles, conference papers, book chapters, and short communications because they did not provide primary experimental data. Additionally, studies that did not specifically focus on *A. salmonicida*, were outside the defined publication period, or lacked sufficient methodological clarity or experimental relevance were excluded. This selection approach ensured the reliability, consistency, and scientific rigor of the included studies [32].

#### 2.4.2. Data Extraction and Synthesis

Data from the included studies were systematically extracted using a standardized data-extraction framework to ensure consistency and comparability. Extracted variables included bibliographic information, host fish species, bacterial strain information where available, phage characteristics, study design, experimental conditions, multiplicity of infection (MOI) or phage dose, environmental conditions, delivery approaches, and reported outcomes, including bacterial reduction, survival rate, and host range. The extracted information was cross-checked for consistency, and discrepancies or ambiguous information were resolved through discussion and consensus. Where information was not reported in the original publication, it was recorded as “not reported” rather than inferred. Additional parameters, including delivery methods and environmental conditions, were recorded where available (Table 1).

To ensure accuracy and reduce bias, the study systematically extracted data, cross-verified it, and discussed and reached consensus to resolve discrepancies. Due to the varied experimental designs, phage types, and measured outcomes, a quantitative meta-analysis was not suitable. Consequently, no pooled analyses, such as risk ratios, odds ratios, or mean differences, were performed to combine data across studies. Instead, a narrative qualitative synthesis categorized the studies based on the phage’s antibacterial efficacy, host specificity, and application in aquaculture systems. The research also facilitated comparisons with other studies, highlighting gaps and suggesting future directions for bacteriophage therapy against *A. salmonicida*.

#### 2.4.3. Quality Assessment

Qualitative assessment of the methodological robustness of the included studies was conducted to evaluate the reliability and translational relevance of the evidence. Given heterogeneity in experimental designs and outcome measures, this assessment utilized a structured appraisal approach instead of a formal risk-of-bias tool. Studies were evaluated across four domains: experimental design (in vitro vs. in vivo), methodological transparency and reproducibility (e.g., MOI and environmental conditions), outcome reporting (e.g., bacterial reduction and survival), and translational relevance (e.g., multi-strain testing and aquaculture applicability), as provided in Table S2.

Based on these criteria, studies were classified as limited, moderate, or strong evidence to facilitate comparison across heterogeneous studies and to summarize the overall strength and translational relevance of the available evidence, as seen in Table 2. In vitro or primarily descriptive studies were generally categorized as low; studies with stronger methodological characterization and partial translational relevance as moderate; and studies including in vivo validation and therapeutically relevant outcomes as strong. No studies were excluded on this basis; instead, this analysis considered differences in evidence strength during interpretation. This qualitative appraisal was not intended to constitute a formally validated risk-of-bias assessment, and the potential subjectivity associated with an author-defined classification was recognized as a limitation of the present review.

### 2.5. Phage Therapy Readiness and Translation Framework (PTRF)

#### 2.5.1. Framework Concept

A PTRF has been proposed to systematically evaluate the translational evidence coverage of bacteriophage therapies targeting *A. salmonicida* based on adaptations of previously published translational readiness and assessment frameworks with minor modifications [135,136,137,138]. This framework was developed as an exploratory, semi-quantitative framework for mapping the breadth of translational evidence reported for *A. salmonicida* phage therapy. Moreover, it is not intended to represent a validated predictive instrument, regulatory assessment, or direct measure of commercial readiness. Although most studies report promising antibacterial efficacy under laboratory conditions, knowledge gaps remain regarding their application in field aquaculture settings. Therefore, the PTRF offers a structured way to assess and compare the biological‚ technical, and implementation factors within one framework.

#### 2.5.2. Five Pillars

The PTRF is composed of five complementary domains: (i) efficacy (E)‚ related to antibacterial activity and in vivo therapeutic efficacy; (ii) host coverage and resistance robustness‚ related to host range‚ strain diversity, and resistance (H); (iii) environmental stability (S)‚ related to stability of phage in different physicochemical conditions; (iv) delivery and formulation (D)‚ related to administration routes‚ protection, and phage release control; and (v) translational feasibility (T)‚ related to field trials‚ scalability, and regulatory approval. Collectively‚ these pillars capture experimentally quantified performance and translational applicability to different practical contexts [139,140,141,142,143].

The five PTRF pillars were chosen as a minimum set of functional criteria for translational phage therapy‚ considering biological efficacy, ecological compatibility, and implementation feasibility. Equal weighting was applied as a deliberate conservative assumption to the pillars to prevent laboratory-centric metrics like efficacy from dominating the phage therapy literature.

#### 2.5.3. Scoring System

Each domain was assigned a semi-quantitative coverage score from 0 to 3: 0 = not investigated or not reported; 1 = preliminary evidence; 2 = intermediate evidence; and 3 = comparatively strong evidence within the evaluated domain. The complete operational criteria for individual domain scores are provided in Table S3. Ambiguous examples were discussed, and consensus was reached. This semi-quantitative approach was used to improve transparency and consistency in applying predefined criteria across heterogeneous studies; however, it does not eliminate subjectivity.

Based on this final score, studies were classified as low (0–5), moderate (6–10), or high (11–15) translational evidence coverage. The five domains were equally weighted, producing a maximum exploratory score of 15. Equal weighting was selected as a transparent and simple methodological assumption to prevent a single domain, particularly laboratory efficacy, from dominating the assessment. However, these weights and classification thresholds have not been empirically validated. PTRF scores should therefore be interpreted as indicators of evidence coverage rather than validated measures of field, commercial, or regulatory readiness.

#### 2.5.4. Application Strategy

The PTRF was applied to all included studies in this systematic review, consistent with established narrative synthesis and evaluation framework methodologies [144]. These studies were scored based on the experimental evidence reported in each publication and a consideration of the strengths‚ weaknesses, and translational nature of the research. This framework allowed for the comparison of results between studies‚ which identified key bottlenecks preventing the translation of phage therapy from bench to aquaculture application.

The PTRF assessment can be interpreted as a structured indication of the breadth of translational evidence reported in each study rather than a definitive prediction of field, commercial, or regulatory readiness. Accordingly, higher scores reflect broader coverage of the evaluated translational domains, whereas lower scores indicate greater evidence gaps within the framework. The resulting scores should therefore be considered comparative indicators within the present evidence base rather than validated measures of phage therapy readiness. In this context, higher PTRF scores indicate comparatively broader translational evidence but do not necessarily imply that a phage intervention is ready for field-scale or commercial application. Table 2 presents the assessment results against the framework.

## 3. Results and Discussion: Research Trends and Experimental Evidence

### 3.1. Publication Trends and Experimental Validation

**Figure 2:**
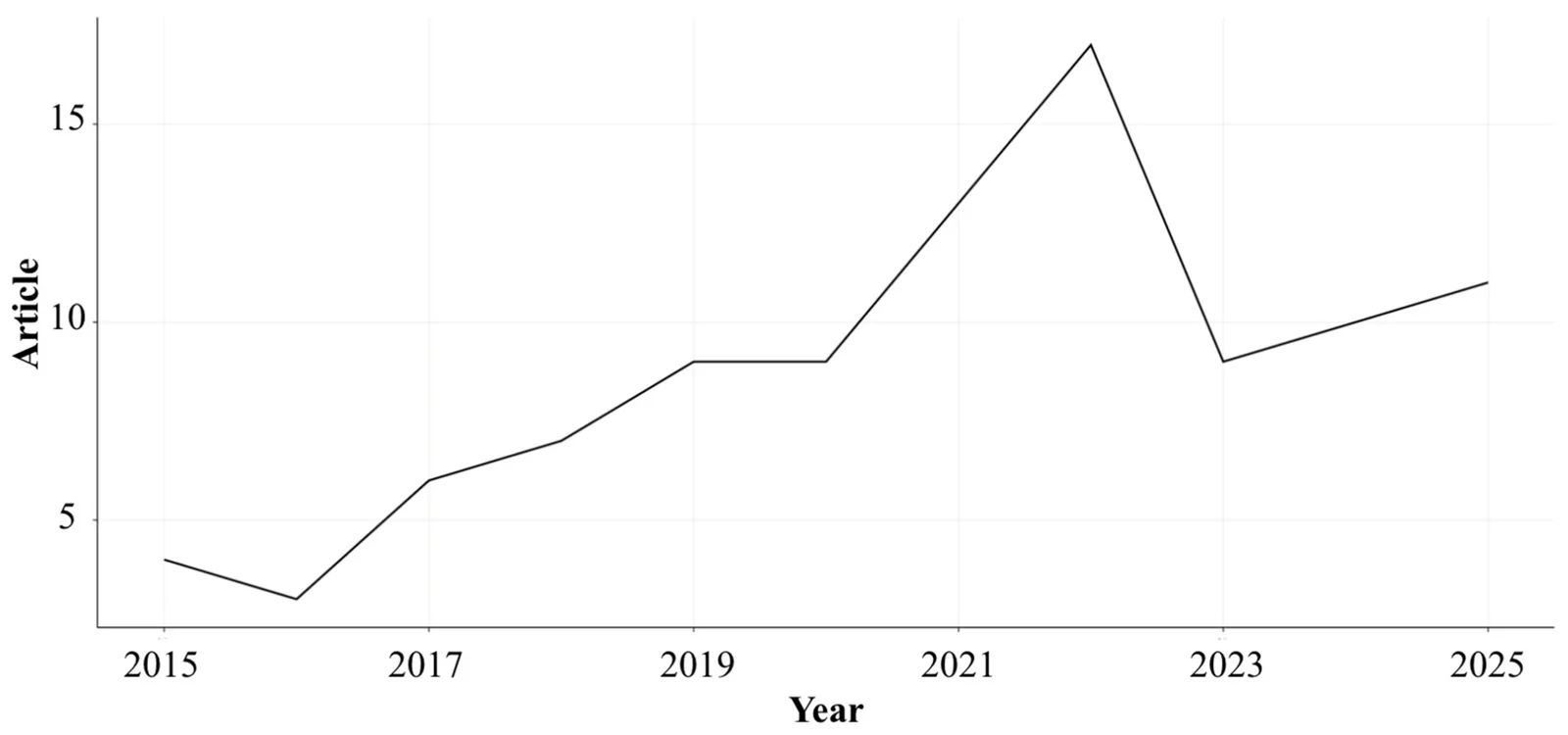
Annual publication trends of the bibliometric dataset (*n* = 108). Data were retrieved from Scopus, Web of Science, and PubMed and analyzed using Bibliometrix/Biblioshiny in RStudio version 2026.01.1.

The bibliometric analysis reveals that the years of publication of experiments related to bacteriophage research associated with *A. salmonicida*, based on keyword-driven retrieval, show a relatively upward trend. The number of publications in this field was relatively low in the early years of 2015–2017‚ while an upward trend can be seen in the number of publications from 2018 onwards (Figure 2). There was a peak of publications in 2022‚ the year with the highest number of publications in the period covered by this study. This pattern could be explained by the increased global awareness of AMR and the renewed interest in phage therapy as a promising alternative method for disease control in aquaculture facilities. However, publication growth should not be interpreted as evidence of therapeutic effectiveness or translational maturity. Evidence on biological efficacy and translational potential therefore came primarily from the systematic synthesis of experimental studies rather than from publication trends.

Importantly, experimental findings from the SLR strongly support this increase in publication activity, with approximately 80% to 90% of included studies reporting significant antibacterial efficacy, including reductions in bacterial load and improved survival rates in infected hosts [25,26,134]. This alignment indicates that the expansion of research activity is driven by validated experimental outcomes.

### 3.2. Leading Countries and Research Institutions

**Figure 3:**
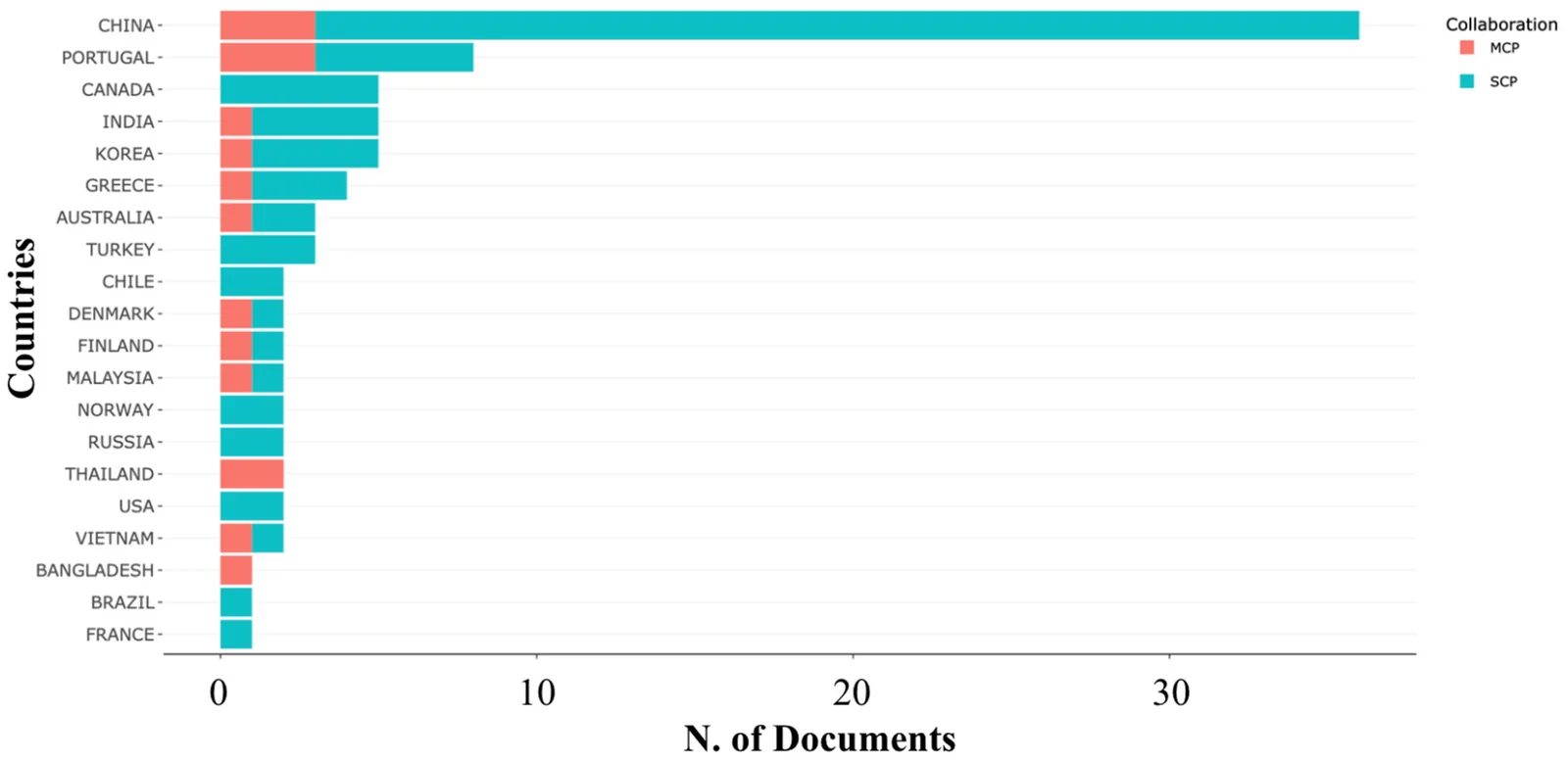
Scientific production by country and collaboration type (SCP vs. MCP) (source: RStudio version 2026.01.1).

The geographical distribution of research activity highlights China as the leading contributor, followed by other major research hubs (Figure 3). Most publications are classified as single-country publications (SCPs) rather than multiple-country publications (MCPs), suggesting that domestic efforts largely drive research and that international collaboration is comparatively limited. Other notable contributors include Portugal, Canada, India, and South Korea, highlighting the growing global interest in phage-based approaches in aquaculture.

**Figure 4:**
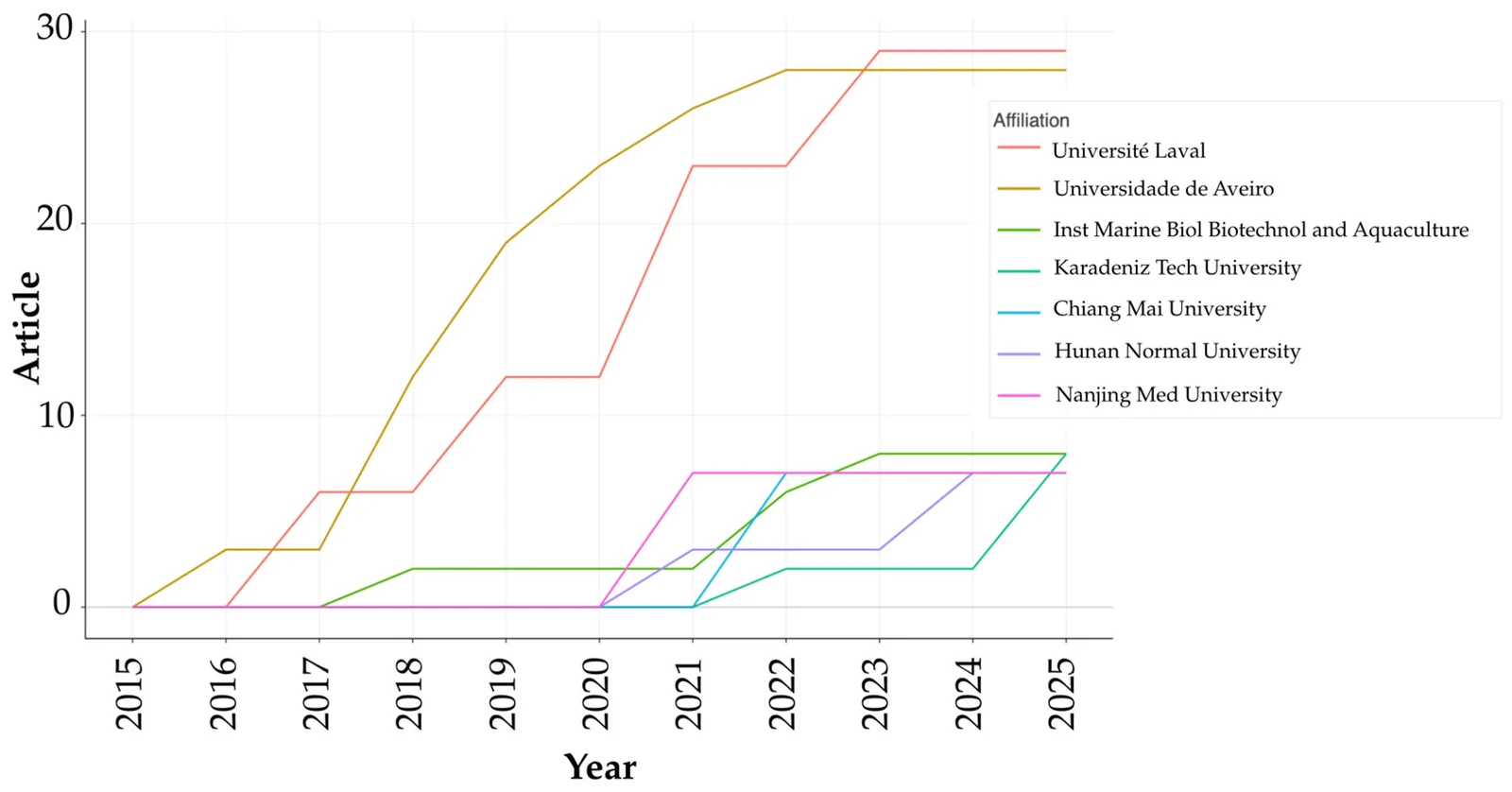
Most productive research institutions in bacteriophage studies are associated with *A. salmonicida* (source: RStudio version 2026.01.1).

At the institutional level (see Figure 4), Universidade de Aveiro and Université Laval are the most productive institutions, followed by several research centers specializing in aquatic microbiology and biotechnology. Overall, research output appears concentrated within a few key institutions, indicating established expertise hubs, while the broader distribution of contributing countries suggests increasing global engagement in this field.

### 3.3. Thematic Evolution and Experimental Support

**Figure 5:**
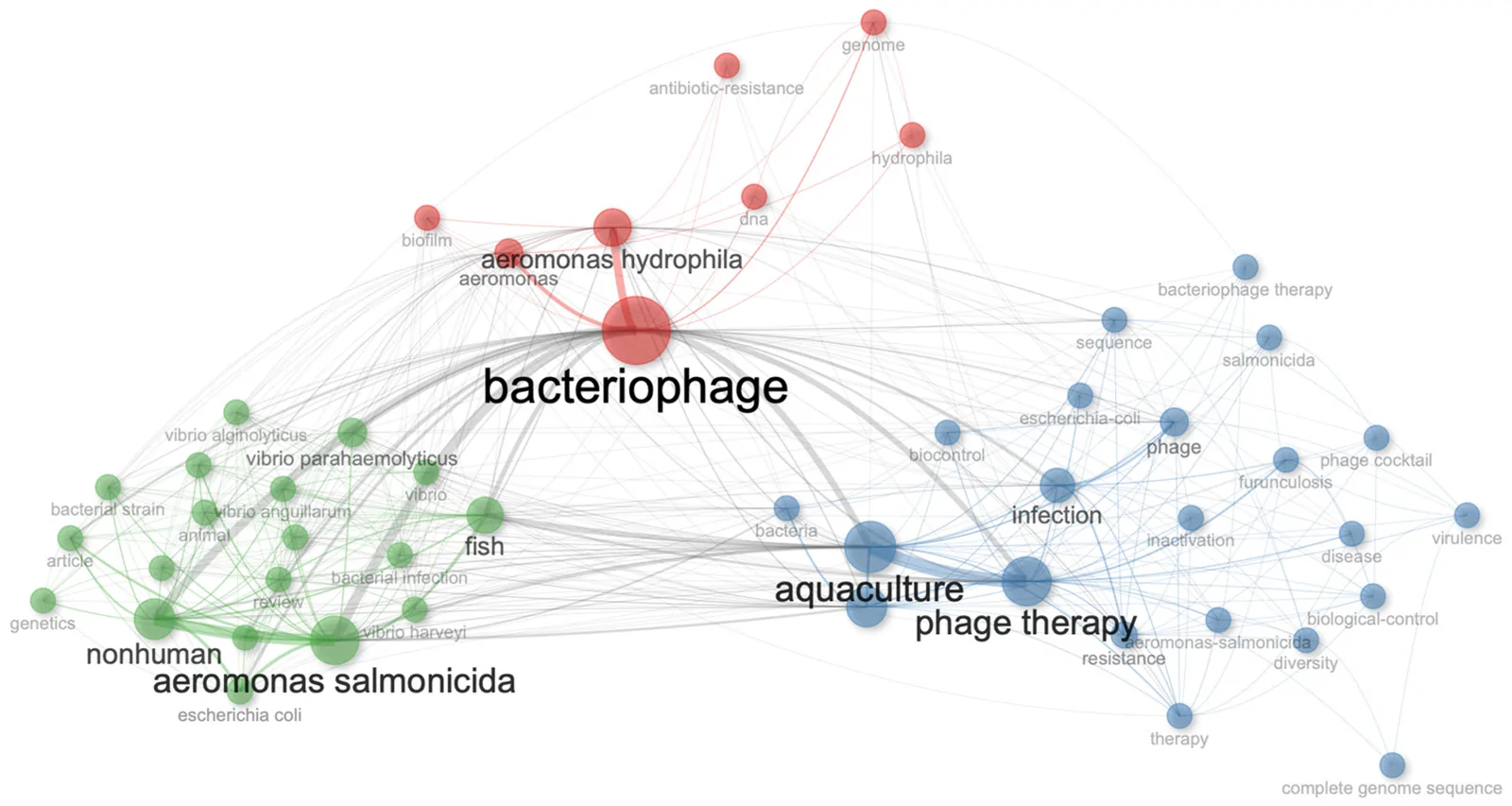
Keyword co-occurrence highlights the interconnectedness of bacteriophage research themes, with strong links among bacteriophage characterization (red), phage therapy and aquaculture applications (blue), host–phage interactions, and pathogen-specific studies (green). Note: size indicates keyword frequency, and link thickness indicates co-occurrence strength (Bibliometrix/Biblioshiny).

The keyword co-occurrence network generated using Bibliometrix/Biblioshiny shows the conceptual structure and major research associated with bacteriophage applications related to *A. salmonicida* (Figure 5). The most studied topics were bacteriophage biology, phage therapy in aquaculture, and studies of *A. salmonicida* pathogens. These clusters illustrate the convergence of basic microbiological research and mechanistic therapeutic approaches.

Bibliometric analysis results show that general bacteriophage studies have shifted over time toward more focused research, leading to trials in aquaculture systems. Early studies primarily focused on phage isolation and characterization, while recent studies have concentrated on therapeutic applications, host–phage interactions, and methods to improve treatment efficacy.

**Figure 6:**
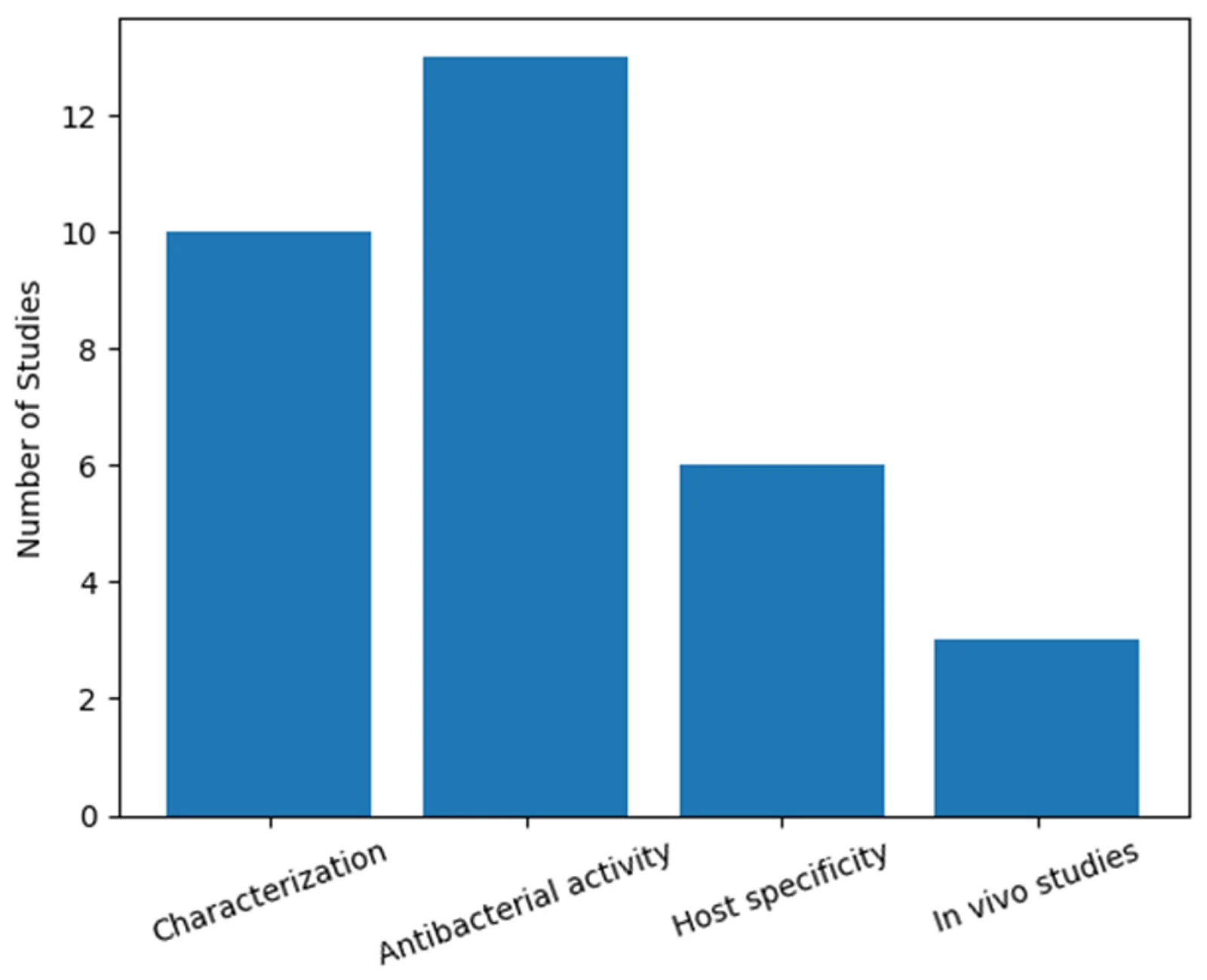
Distribution of the main research topics among the 22 included studies. Categories are non-mutually exclusive, as a single study could address more than one research topic; therefore, the sum of category counts exceeds the total number of included studies (*n* = 22).

As shown in Figure 6, the SLR confirms the prevalence of the mentioned themes. Experimental studies explicitly related to phage issues screened across all four categories‚ including 10 studies on phage characterization‚ 13 on antibacterial activity assays‚ six on host specificity‚ and three on fish in vivo studies. Experimental methods included both in vitro bacterial reduction and in vivo animal and bacterial infection models. All but two of the studies (17 out of 22; ~77.3%) reported important antibacterial potency‚ suggesting that the new trends of this line of research are worthy of experimental exploration.

The convergence between thematic evolution based on keyword analysis and experimental outcomes suggests the field is moving from decentralized exploratory research landscapes towards application-focused studies. This transition underscores the growing potential of bacteriophage therapy as a cost-effective, sustainable method to combat *A. salmonicida* infections in aquaculture, particularly as an antibiotic replacement.

### 3.4. Application of the Phage Therapy Readiness and Translation Framework (PTRF)

**Figure 7:**
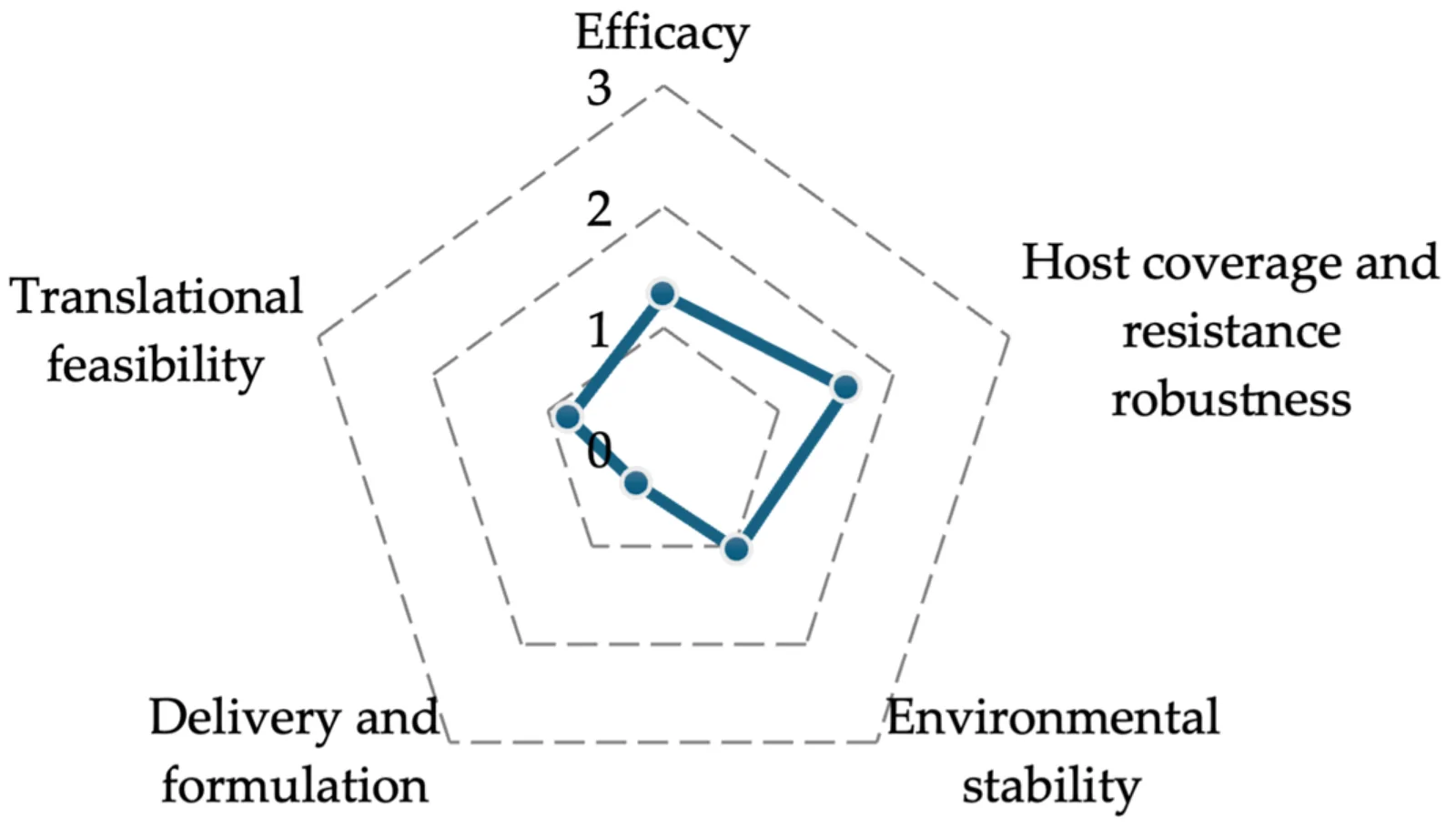
Mean PTRF domain scores (0–3) across the 22 included studies. The five domains represent different dimensions of translational evidence coverage.

**Figure 8:**
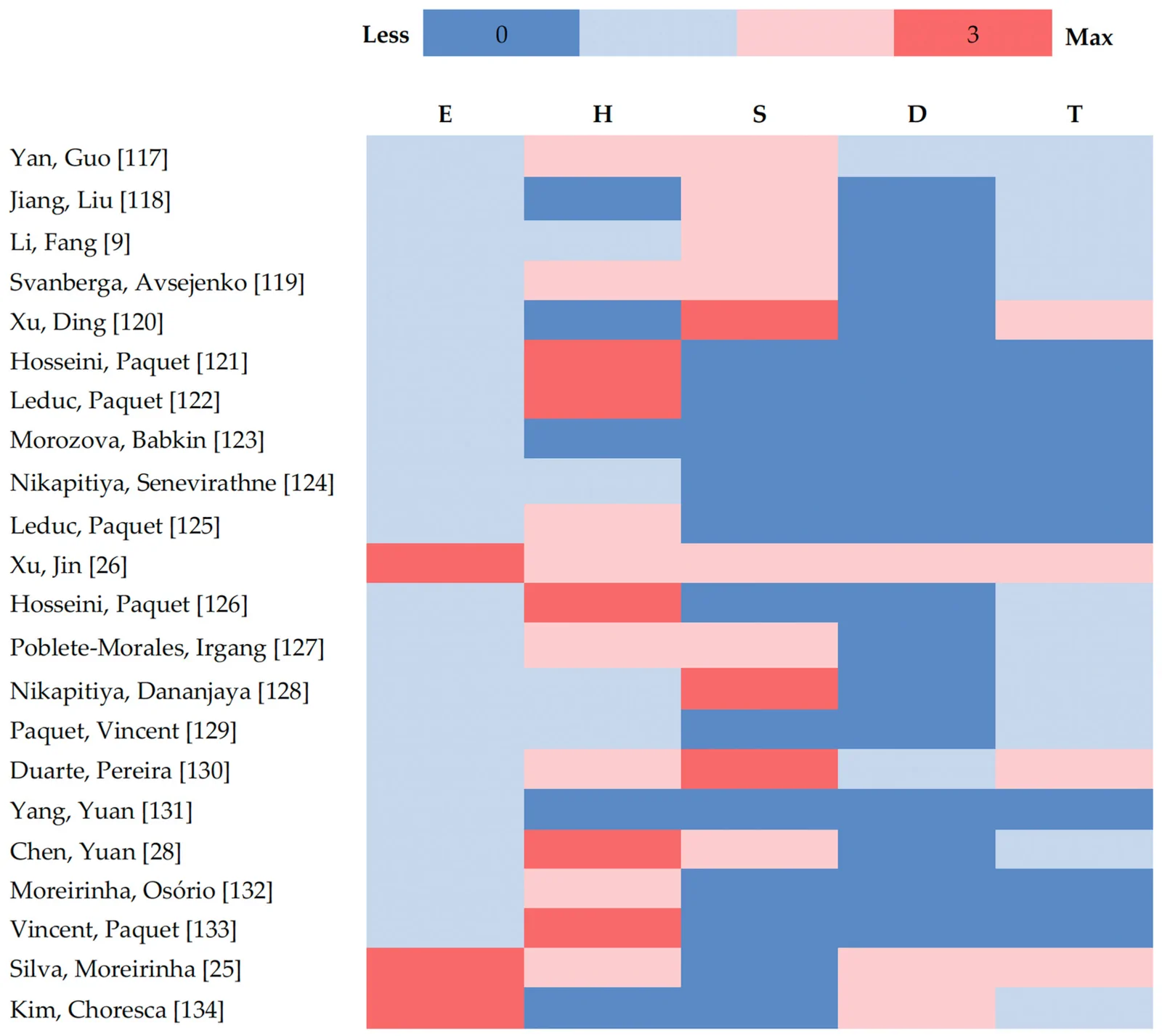
Heat map of PTRF domain scores across the included studies. E, efficacy; H, host coverage and resistance robustness; S, environmental stability; D, delivery and formulation; T, translational feasibility. Higher scores indicate broader translational evidence coverage within the respective domain [9,25,26,28,117,118,119,120,121,122,123,124,125,126,127,128,129,130,131,132,133,134].

The integrated quality and PTRF-based evaluations of the included studies are presented in Table 2. Using the PTRF assessment rubric‚ most studies in our review were found to fall into the low translational evidence coverage level‚ with some in the moderate translational evidence coverage level and none in the high translational evidence coverage level. Phage performance pillar studies were the highest overall‚ especially those that reported strong in vitro lysis or increased in vivo survival. Delivery and formulation and translational feasibility were the lowest overall. This imbalance is further illustrated in Figure 7, which presents the mean PTRF score across all included studies (*n* = 22). The study-level variation in these domains is further visualized in the PTRF heat map (Figure 8), allowing differences in the breadth of evidence across individual studies to be readily identified.

A study with broader translational evidence coverage was reported by Xu, Jin [26]. This study integrated in vitro characterization, which was followed up with in vivo therapeutic evaluation, and improved host survival of infected fish with defined delivery strategies. While none achieved high translational evidence coverage by covering the highlighted gaps of wide host coverage‚ field-scale validation‚ and production or regulatory feasibility‚ they are the strongest studies to date. Other studies with acceptable to good efficacy and poor delivery and translational feasibility, including Vincent, Paquet [133], Moreirinha, Osório [132], and Yang, Yuan [131], show that the field remains efficacy-driven and translation-limited.

Studies with high host coverage and resistance robustness‚ including Hosseini, Paquet [121], Leduc, Paquet [122] and Vincent, Paquet [133], provided important mechanistic insights into receptor binding and resistance development but had limited direct therapeutic implications. Likewise‚ Xu, Ding [120] and Poblete-Morales, Irgang [127] performed well in environmental stability assessments but were limited by their performance in the other pillars‚ suggesting that neither can independently support translational development‚ even as they inform formulation strategy. Ultimately‚ the PTRF states that the scientific basis is present for phage therapy against *A*. *salmonicida* but that there are translational evidence gaps.

### 3.5. Integrated Synthesis of Evidence

Bibliometric‚ systematic review‚ and PTRF results indicate an imbalance in research interest within the field of bacteriophage therapy against *A. salmonicida*. The bibliometric analysis provides contextual information on the content and development of the research field. In contrast, the systematic literature review provides the principal evidence base for evaluating experimental efficacy and translational gaps. Research output appears to be growing and is largely promising in vitro‚ with several prior successes in vivo; however, translation to the clinic has been limited. The PTRF results indicate that efficacy and‚ to a lesser extent‚ host coverage are well developed‚ but environmental stability‚ delivery, formulation‚ and translational feasibility are less well developed (Table 3).

Table 3 demonstrates substantial heterogeneity in the evidence supporting phage therapy against *A. salmonicida*. Most studies were limited to in vitro or genomic investigations, such as Yan, Guo [117] and Li, Fang [9], resulting in limited translational coverage despite demonstrating lytic activity. Similarly, studies by Hosseini, Paquet [121] and Chen, Yuan [28] evaluated phage cocktails in vitro but lacked in vivo validation. In contrast, Xu, Jin [26] and Silva, Moreirinha [25] provided strong evidence through controlled in vivo experiments, although both remained limited by the absence of field validation. Kim, Choresca [134] also demonstrated in vivo efficacy but reported limitations related to phage persistence. Overall, studies with in vivo evidence generally showed broader translational coverage than in vitro-only studies, while formulation, delivery, environmental stability, scalability, and field validation remained insufficiently addressed. These findings support using PTRF as a structured measure of evidence breadth rather than efficacy alone.

However, due to the majority of published work showing biological activity‚ and large variation in experimental design and reporting of results‚ it has been difficult to compare and contrast different approaches and devise effective therapeutic strategies. In general‚ phage therapy has shown promise but has not progressed sufficiently for application in aquaculture‚ and more translational research is required to apply phage therapy in the field.

### 3.6. Practical Aspects and Findings from A. salmonicida Phage Therapy Studies

#### 3.6.1. Mechanisms of Phage Therapy Against *A. salmonicida*

**Figure 9:**
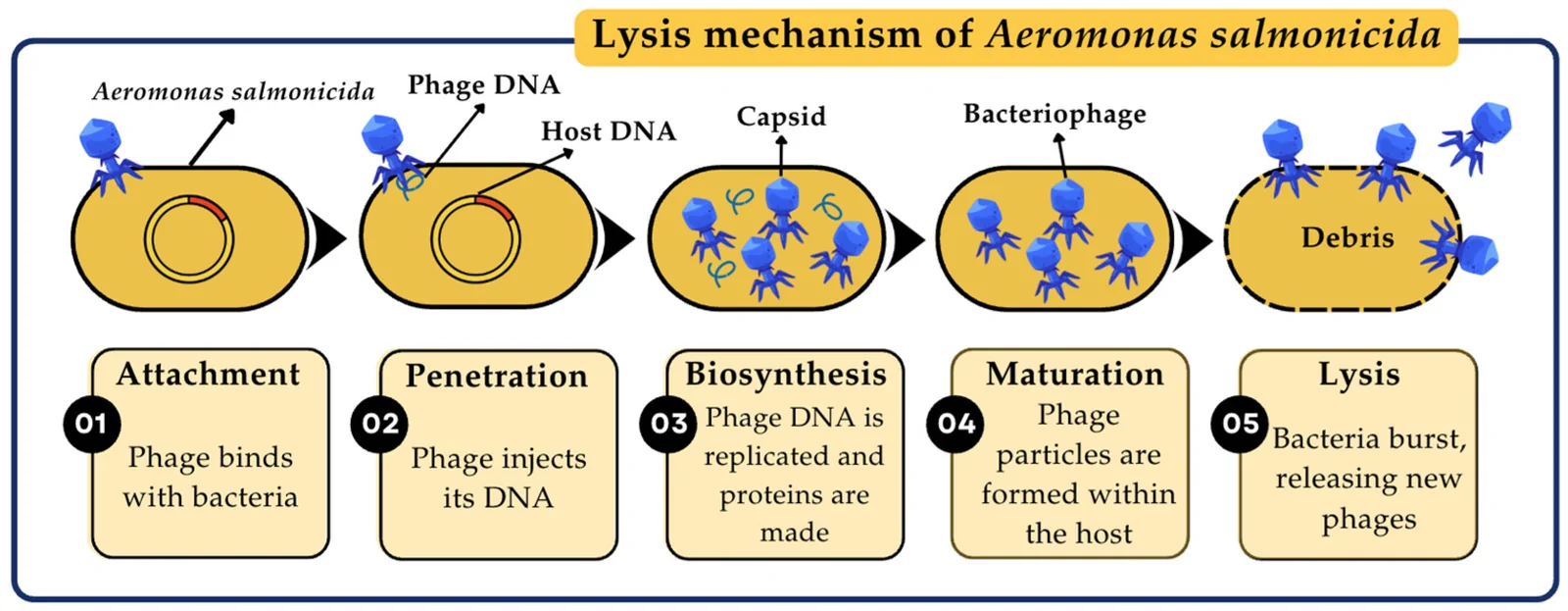
Schematic representation of the lytic bacteriophage infection cycle, including attachment (adsorption), penetration/genome injection, intracellular replication and assembly, and host-cell lysis with release of progeny phages.

The effectiveness of bacteriophage therapy against *A. salmonicida* primarily depends on the phage–host interaction. This interaction results in the biological characterization of the phage, such as its host range, resistance development, and environmental stability. (Figure 9). These characteristics form the biological basis for the translational barriers defined by the PTRF analysis.

A. Phage adsorption and host specificity

The initial step in phage infection is adsorption or attachment, in which the bacteriophage binds to specific receptors on the bacterial cell surface [145]. These receptors include lipopolysaccharides (LPSs), outer membrane proteins, flagella, or capsular polysaccharides [146,147]. Phage recognition of these receptors is mediated by specialized structures, such as tail fibers or receptor-binding proteins (RBPs) located on the phage capsid or tail [148,149]. As they are highly variable‚ this lowers the average host coverage pillar of the PTRF (Figure 7). This specificity necessitates strain-level screening and indicates that phage cocktails with diverse bacterial receptor recognition might expand host range and decrease the chance that changes, or loss of one receptor will make bacteria resistant to all cocktail components.

In *A. salmonicida*, variations in bacterial surface structures can influence the susceptibility of bacterial strains to different phages [129,150,151]. The presence of the A-layer (surface layer/S-layer) of *A. salmonicida* reduces plaque formation or alters plaque morphology in phages that use hidden outer membrane receptors [129]. The specificity of receptor recognition explains why many bacteriophages exhibit a narrow host range, infecting only certain bacterial strains [152,153]. This specificity can be advantageous in aquaculture applications because phage therapy selectively targets pathogenic bacteria while preserving beneficial microbial communities in the aquatic environment, but it may not provide broad protection against multiple strains of the target bacterium.

B. Lytic activity and antibacterial efficacy

The antibacterial activity of bacteriophages is mainly due to their lytic activity; once phages infect and replicate in a susceptible bacterial cell‚ they will lyse the host cell [154,155]. The lytic cycle results in a rapid decline in the number of bacteria‚ accounting for the high efficacies achieved [156]. In vitro‚ phage efficacy is mostly a measure of the efficiency of phage multiplication in vitro [104,157].

However, the association between lytic activity and therapeutic effectiveness may be complex; in vitro lysis is indicative of potential biological activity‚ although in vivo results may vary under different aquaculture conditions [120,125]. Next‚ invariant strain diversity‚ infection dynamics‚ and environmental variability can all reduce efficacy [158]. Thus, the discovery of these processes within the PTRF structure explains why efficacy scores are moderate‚ even when translational evidence coverage is low.

C. Bacterial resistance and phage–host coevolution

The coevolution between bacteriophages and *A. salmonicida* is intimately linked to the bacterium’s endogenous mechanisms to defend against phage infection and can determine the success or failure of phage therapy efforts [159,160,161]. Therefore, phage resistance has become a major limitation to the durability of bacteriophage therapy against *A. salmonicida* [126,129]. Initial bacterial reduction does not necessarily predict sustained therapeutic efficacy because resistant subpopulations may emerge during continued phage exposure [162,163]. As discussed above, resistance can occur through alterations of surface receptors [151], including LPS and A-layer components, as well as through intracellular defense mechanisms such as restriction–modification and CRISPR–Cas systems [164,165]. Prophage-associated immunity and population-level heterogeneity may further reduce susceptibility to therapeutic phages [121,126]. This has led to counter-adaptations in bacteriophages, such as diverse receptor-binding proteins‚ genome-wide remodeling‚ and the evolution of anti-defense proteins, as presented in Table 4.

Furthermore, resistance is not simply an endpoint of lab-based characterization but may influence bacterial population structure and therapeutic response [166,167]. Consequently‚ this study recommends that the translational development of phage therapy include longitudinal testing for resistance development‚ testing against genetically diverse *A*. *salmonicida* isolates‚ and assessing the impact of resistance-associated phenotypes on fitness and associated changes in bacterial virulence to inform the clinical use of phage therapy. Complementary receptor usage and resistance profiles should be considered in phage selection and cocktail design.

D. Evolutionary dynamics

Interactions between bacteriophages and *A. salmonicida* involve swift coevolution, where the host quickly develops resistance to the phages, and the phages, in turn, evolve strategies to overcome this resistance [159,160,161]. In aquaculture, coevolution plays a crucial role because of the high host densities, frequent exposure of hosts to pathogens, and environmental stressors that often drive quick diversification in hosts and selection pressures from phages [168]. As a result, effective phage therapy relies on the initial lytic properties of phages and their capacity to stay active as bacteria develop resistance [169]. Additional general phage resistance mechanisms, such as receptor modification, restriction–modification, and CRISPR-associated phage immunity, are well-defined and are thought to pose an important burden to long-term phage therapy [170].

The host range of phages infecting *A. salmonicida* is variable among isolates‚ with surface properties and the lifestyle of the bacteria influencing susceptibility [119,125,133]. In a large study of host range‚ some phages infected more than 44 out of 65 tested isolates‚ while others only infected a handful. Overall‚ 44RR2.8t.2 and SW69-9 had the broadest range within that panel and together covered all *A. salmonicida* subsp. salmonicida strains. These findings point to a coevolutionary response‚ whereby receptor and strain diversity may make the selection of resistant subpopulations likely unless therapy employs multiple complementary phages [133].

E. Surface-mediated resistance

The most well-defined phage resistance mechanism in *A*. *salmonicida* is through alteration of surface receptors that phages attach to when adsorbing to a bacterial surface [129]. The lipopolysaccharide (LPS) structure and the A-layer/S-layer of *A*. *salmonicida* are both involved in phage recognition [151]. Most notably‚ a recent review of *A*. *salmonicida* biology also noted that while LPS lipid A was the receptor component for phage 55R-1 [129]‚ the receptor component for phages TP446 and SW69-9 had been reported as its A-layer. Taken together‚ this suggests that *A*. *salmonicida’s* change in receptor composition is a likely explanation for its phage resistance [115]. 

Experimental selection of *A*. *salmonicida* mutants resistant to phage has shown that receptor-based resistance can result in large changes to the expression of proteins and in cell envelope-associated characteristics of the bacterium‚ suggesting that receptor-mediated resistance may not be an effective long-term strategy [132]. Phage that is effective at the start of treatment may become ineffective if resistant strains with altered receptor structures arise [171,172].

F. Intracellular defense systems

In addition to surface resistance mechanisms, *A. salmonicida* has deployed intracellular defense systems that act post-phage entry to block replication and dissemination [132]. Most notable among these are restriction–modification (R–M) systems and CRISPR–Cas adaptive immunity, both of which play key roles in protecting bacterial cells against foreign genetic material [164,165].

Restriction–modification systems can cleave foreign (i.e., unmethylated) invading phage genomes while leaving their own protected by specific methylation marks [173,174]. Bacterial restriction–modification systems provide a fast and effective barrier to phage infection‚ but phages can escape these systems by either methylating their nucleic acid genomes or producing anti-restriction proteins [175,176].

Other bacterial adaptive immunity systems include the CRISPR-Cas systems. Here‚ spacers derived from phages that had previously infected the bacteria are incorporated into the bacterial genome at particular sites and used to identify and digest phage DNA with the same sequence in subsequent infections [177]. CRISPR-Cas systems have been identified in species of *Aeromonas*‚ although their distribution and activity in the many strains of *A*. *salmonicida* have been variable [178,179].

G. Population-level defense mechanisms

Population-level defense mechanisms‚ such as heterogeneous bacterial populations and heterogeneous phage-resistance mechanisms between subpopulations‚ may also play a critical role in controlling the overall dynamics of bacteriophage and *A. salmonicida* interactions, as they can confer the ability of the population to buffer itself from phage-induced death and therefore persist in the environment [180,181].

Prophage-mediated immunity is one of the strongest modes of antibacterial immunity at the community level: lysogenic bacteria that harbor an integrated prophage may confer superinfection exclusion, preventing the adsorption and delivery of therapeutic phages and thus limiting the efficacy of phage therapy [182]. Prophage elements in *A. salmonicida* have been associated with reduced susceptibility to certain lytic phages [121,126].

**Table 4:** Bacterial defense mechanisms and corresponding phage counter-adaptations in *A. salmonicida*.

Bacterial Defense Mechanism	Mode of Action	Evidence in *A. salmonicida*/Aquatic Pathogens	Phage Counterstrategy	Key Implication for Therapy
Receptor modification	Alteration or loss of surface receptors (e.g., LPS, A-layer) prevents phage adsorption	Observed in *A. salmonicida* phage-resistant isolates; associated with LPS and A-layer variation [115,129]	Tail fiber mutations, host-range expansion	May reduce phage efficacy; supports use of phage cocktails targeting multiple receptors
Restriction–modification (R–M) systems	Cleavage of foreign (unmethylated) phage DNA inside the host cell	Identified in bacterial defense systems and aquatic pathogens [170,183,184]	DNA methylation, anti-restriction proteins, genome modification [185,186]	Requires selection of phages capable of bypassing intracellular defense mechanisms
CRISPR–Cas systems	Adaptive immunity via spacer acquisition and targeted degradation of phage DNA	Present in *Aeromonas* spp.; heterogeneous distribution in *A. salmonicida* [178,179]	Anti-CRISPR proteins, protospacer mutations [185]	Indicates need for strain-specific phage selection
Abortive infection (Abi)	Infected cell undergoes programmed death to prevent phage replication	Suggested in aquatic bacterial defense systems [170]	Anti-Abi genes, lifecycle modulation [185,187]	Reduces phage amplification; may limit therapeutic efficiency
Superinfection exclusion (Sie)	Prevents secondary infection by additional phages in already infected cells	Associated with prophage elements in aquatic bacteria [170]	Receptor bypass, timing-based infection strategies	Important for designing compatible phage cocktails
Surface shielding (biofilm/EPS)	Biofilm matrix blocks phage penetration and adsorption	Biofilm formation in *A. salmonicida* reduces susceptibility [188]	Phage-encoded depolymerases degrading EPS [189,190]	Supports use of biofilm-degrading phages for improved efficacy
Prophage-mediated immunity	Lysogenic phages provide resistance to superinfection	Observed in *A. salmonicida* strains carrying prophages [170]	Use of unrelated lytic phages, cocktail design	Highlights importance of excluding lysogenic phages
Phase variation	Reversible switching of surface structures affecting receptor availability	Suggested in *Aeromonas* spp. affecting susceptibility [181,191]	Broad host-range phages, adaptive cocktails	Contributes to dynamic resistance; requires flexible therapy design
BREX system	Epigenetic defense preventing phage replication without DNA cleavage	Identified in bacterial defense islands [185]	Phage DNA mimicry, anti-BREX proteins [185]	Emerging resistance mechanism requiring genomic screening
DISARM system	Multi-gene defense targeting foreign DNA	Present in bacterial defense clusters [170]	Genome modification strategies	Requires advanced phage adaptation strategies
CBASS system	Detection of infection triggers cell suicide to block phage replication	Emerging defense in bacteria [185]	Anti-CBASS proteins	May reduce phage propagation efficiency
Defense islands	Genomic clusters combining multiple defense systems	Common in aquatic pathogens and defense systems [170,192]	Multi-target phage strategies, engineered phages	Emphasizes need for multi-phage and engineered approaches

#### 3.6.2. In Vitro Study Efficacy

**Figure 10:**
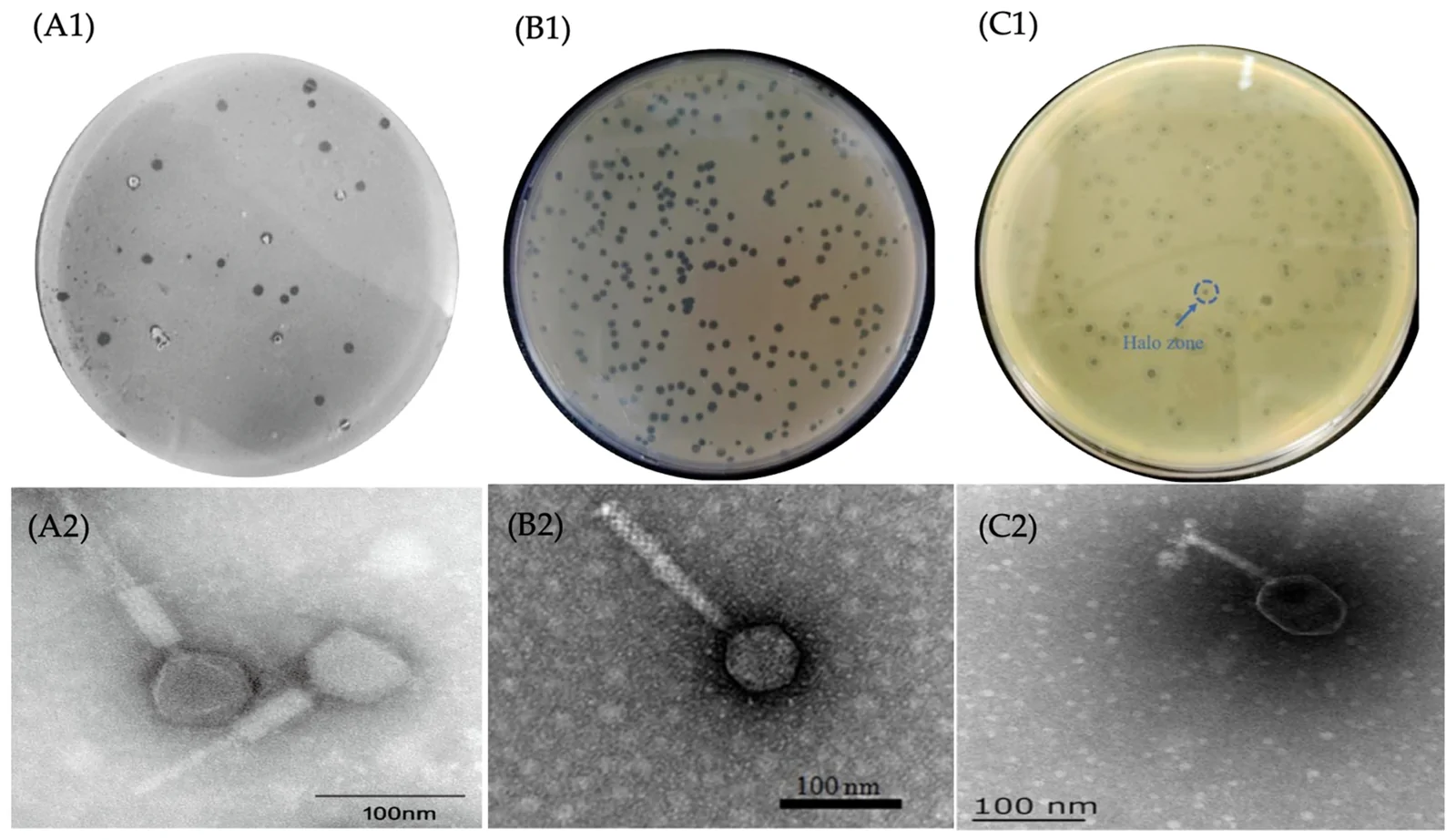
Plaque and virion morphology of *A. salmonicida* phages ASG01 [9], ASP-1 [128], and TSW001 [117]. (**A1**) Plaque morphology of phage ASG01 on a double-layer agar plate; (**A2**) virion morphology of ASG01 observed by transmission electron microscopy (TEM), scale bar = 100 nm. (**B1**) Plaque morphology of phage ASP-1 on a double-layer agar plate; (**B2**) virion morphology of ASP-1 observed by TEM, scale bar = 100 nm. (**C1**) Plaque morphology of phage TSW001 on a double-layer agar plate; (**C2**) virion morphology of TSW001 observed by TEM, scale bar = 100 nm.

Many in vitro studies have reported a high antibacterial activity of bacteriophages against *A. salmonicida* and a substantial decrease in bacterial load, as seen in Figure 10 [26,117,128]. These studies provide important evidence of phage isolation, host specificity, adsorption, lytic activity, resistance mechanisms, and environmental stability. However, in vitro efficacy should be regarded as evidence of biological activity rather than direct evidence of therapeutic effectiveness under aquaculture conditions. This may explain the high values of the efficacy pillar of the PTRF analysis (Table 2).

Two major limitations of in vitro results are as follows: first‚ in vitro systems are simplified and may be less relevant for aquaculture‚ where other important factors such as strain heterogeneity‚ biofilm formation, co-infections, and environmental differences may be more prominent, leading to overprediction of the therapeutic efficacy; and second‚ as shown by the PTRF results‚ papers that are based solely on in vitro studies tend to score high on efficacy and host coverage and resistance robustness but low on overall translational evidence coverage because of limitations on the translational aspect (Figure 7).

#### 3.6.3. In Vivo Study

Only three of the 22 included studies reported in vivo assessment in fish (Table 1). These studies provide a higher level of biological validation because they examine phage performance within a living host. Nevertheless, the small number of studies, differences in fish species, phage preparations, administration routes, and experimental conditions prevent generalization to commercial aquaculture and highlight the critical need for further in vivo-based studies in the future.

The strongest in vivo evidence was reported by Xu, Jin [26], who evaluated the lytic phage (vB_AsM_ZHF) tested in vitro and in vivo on turbot (35.0 ± 5.0 g) under controlled laboratory conditions in a glass tank (60 L of seawater)‚ showing a reduction in mortality and bacterial load. These data from the study make it the most translationally relevant in vivo study in this collection because it directly links antibacterial efficacy to host-level therapeutic benefit under an applied delivery modality.

Silva, Moreirinha [25] indicated that lytic phage therapy considerably decreased mortality and infection in *Senegalese sole*‚ as well as providing evidence that phage therapy can remain active in living fish and can help improve disease outcomes in vivo in fish, as well as in vitro in tubes.

The earliest in vivo study in the dataset is Kim, Choresca [134], where phage PAS-1 was studied in *O. mykiss.* The study reported varied outcomes, including increased survival and phage clearance within 200 h, as well as a host immune response. This study is promising not only for its therapeutic success but also for providing initial evidence on phage persistence and host biological response.

Taken together, these studies provide preliminary evidence that selected *A. salmonicida* phages can improve disease outcomes under controlled experimental conditions. However, the small evidence base and substantial heterogeneity in host species, bacterial challenge models, phage dose, administration route, and outcome measures preclude extrapolation to commercial aquaculture. These findings should therefore be regarded as preclinical proof of concept rather than evidence of established field effectiveness. Rather, they provide comparatively stronger translational evidence than studies restricted to in vitro characterization, while important gaps remain regarding field-scale efficacy, standardized delivery protocols, manufacturing, environmental safety, and regulatory requirements.

Ultimately‚ this systematic review and PTRF analysis show that while phage therapy has biological efficacy against *A*. *salmonicida* in vitro and in vivo‚ its application in aquaculture is limited. Research should shift to more field-relevant testing‚ standardization of experimental designs and protocols‚ and consideration of delivery methods and environmental factors associated with phage therapy in aquaculture to enable further advancements in this field.

To compare therapeutic outcomes and application strategies across studies, the key findings are summarized in Table 5. The expanded synthesis finds that while antibacterial efficacy is consistently reported across studies, only a few of the studies used in vivo validation and practical administration routes (immersion and feed). Future research could address a number of limitations to date, including standardization of challenges‚ multiple *A*. *salmonicida* strains‚ development of phage formulations for long-term storage and environmental stability‚ and pilot plant-scale tests under commercial aquaculture conditions. These factors will need to be addressed before phage therapy can be translated from proof-of-concept to the clinic.

#### 3.6.4. Phage Cocktails: Enhancing the Efficacy Against *A. salmonicida*

A. Limitations of single-phage therapy

Furthermore‚ 22 of the studies included in the systematic literature review were single-phage applications against individual or multiple bacterial isolates. This does not reflect the strain heterogeneity occurring within aquaculture systems. While they show strong in vitro antibacterial activity and, in some cases, improved in vivo survival‚ limited host coverage and potential for phage resistance development largely limit the potential for these phages to be used.

This pattern is also reflected in the PTRF analysis, in which host coverage and resistance robustness received higher scores than efficacy, while delivery and translational domains showed comparatively lower evidence coverage (Figure 7). Mechanistic studies [129,133] included in the review have also shown that *A*. *salmonicida* can easily evolve resistance through receptor modifications‚ prophage-mediated immunity‚ and intracellular defense mechanisms‚ pointing to the clear limits of using single-phage treatments in terms of durability.

B. Concept and advantages of phage cocktails

To overcome the shortcomings of single-phage therapeutics, researchers have also begun investigating multiphage therapeutics, i.e., mixtures of bacteriophages active against a common pathogen [28,193]. Combining phages with overlapping host ranges creates a phage cocktail that can significantly enhance antibacterial effectiveness by targeting different receptors [194].

Phage cocktails might offer various advantages for *A. salmonicida* [195]. Expanded host range: multiple phages improve the likelihood of infecting various bacterial genomes. Reduced resistance development: bacteria must acquire multiple mutations to resist all phages in the cocktail. Synergistic antibacterial activity: co-infection with phages enhances bacterial lysis through combined effects. Increased treatment stability: cocktails stay effective even if some bacterial strains develop resistance to one phage.

Current studies have highlighted the potential of phage therapy as a sustainable alternative to antibiotics in aquaculture due to the increasing antimicrobial resistance of aquatic pathogens [31,196].

C. Experimental evidence supporting phage cocktails

Few empirical studies have assessed the efficacy of phage cocktails against *A. salmonicida*, though available studies show clear advantages over single-phage approaches. A minority of studies on *A. salmonicida* phage therapy have directly tested multi-phage treatments. Hosseini, Paquet [121] found that a four-phage cocktail could maintain extended bacterial suppression compared to any individual phage; they also suggested that prophage-mediated bacterial resistance may limit phage therapy efficacy. Chen, Yuan [28] also found that combinations of two phages showed synergistic antibacterial effects in vitro but not in aquaculture-associated settings.

Additional indirect evidence for the need for cocktails comes from significant variability in phage host range across strains [133], along with their generally narrow host range [119]. This suggests that a single phage is unlikely to target multiple strains effectively. However, existing research is limited by its focus on in vitro studies, diverse methodologies, and a lack of data on resistance dynamics.

Phage cocktails appear promising in proof-of-concept studies yet have not been tested thoroughly enough for field application. Future research in aquaculture should focus on standardization‚ multi-strain testing‚ and in vivo/field testing.

D. Challenges and drawbacks

Although phage cocktails offer wider coverage and may reduce the risk of resistance to a single phage, adding more phages does not necessarily improve results [197,198]. Phages may compete for the same receptors‚ adsorb to host cells or propagate at different rates‚ or interfere with one another when used in combination [198,199]. As a result‚ the activity of a cocktail cannot always be predicted from the activity of its components. Even when phage cocktails are used, *A. salmonicida* may also evolve resistance through mechanisms such as receptor modification, restriction–modification systems, or CRISPR–Cas immunity [200,201]. However, combining multiple phages targeting different bacterial receptors can significantly reduce the likelihood of resistance and improve treatment effectiveness [126].

Another drawback is the low genetic diversity of *A*. *salmonicida* strains isolated from aquaculture facilities‚ which may result in different degrees of susceptibility to individual phages [202]. Thus‚ it is important to continue the search for and characterization of phages against *A*. *salmonicida*. For example, the podophage JELG-KS1 was lytic to this bacterium‚ though it exhibited a narrow host range; thus‚ multi-phage cocktails may be useful [119]. Environmental factors, such as temperature‚ pH‚ and exposure to ultraviolet light, might also affect phage therapy and can influence the stability and persistence of phage in aquaculture systems [18,203]. Thus‚ it may be necessary to optimize phage delivery strategies and storage formulations to ensure that phage levels are adequate in fish farms [204,205].

Furthermore, standardizing cocktails introduces additional manufacturing and quality-control challenges because different phages have varying stabilities, yields, purification, and storage requirements, complicating efforts to maintain consistent proportions in the mixture [206,207,208]. Changes in bacterial populations may also influence the relative potency of the various things being combined [198]. Thus, rational cocktail design may involve considering complementary receptor usage and host-range coverage, resistance profiles, phage compatibility, and the stability of the resulting cocktail during preparation, rather than just maximizing phage diversity [209].

#### 3.6.5. Environmental Stability and Delivery of Phages in Aquaculture Systems

The success of phage therapy in aquaculture depends not only on phage lytic activity but also on their stability and ability to reach the target host. Aquaculture environments are complex ecosystems with diverse physical factors, such as pH and temperature, as well as chemical conditions, that can influence phage stability, infectivity, and overall efficacy [119,210].

A. Environmental factors affecting phage stability

Another important translational evidence gap in phage therapy for aquaculture concerns environmental stability. This gap is reflected in the relatively low PTRF score (1.05) for the environmental stability domain (Figure 7). The SLR identified limited evidence examining how temperature, pH, salinity, UV exposure, organic matter, and other environmental conditions affect phage persistence and infectivity, with even fewer studies linking these factors to therapeutic outcomes. Among these, UV radiation is the most significant factor because phages are rapidly inactivated by sunlight [211,212]. Experimental evidence by Duarte, Pereira [130] also showed that UV radiation significantly reduced *A. salmonicida* phage viability, with reported declines of ~1.3 log PFU mL^−1^ under UV-B and ~2.7 log PFU mL^−1^ under direct sunlight.

Moreover, capsid structure and success of infection can also be affected by the temperature‚ the pH‚ and the characteristics of organic matter and suspended particles such as sediment in a medium [120,213,214]. Most studies‚ however‚ have looked at these factors separately‚ and not under aquaculture conditions.

Therefore, environmental assessment of phage therapy should extend beyond short-term measurements of phage stability under different temperature, pH, salinity, and UV conditions. Repeated or large-scale administration may alter phage abundance and distribution within aquaculture systems and surrounding aquatic environments [215]. The persistence of therapeutic phages may also depend on organic matter, suspended particles, microbial communities, and environmental turnover [216,217].

Additionally, the impact on non-target microbial communities and the shifts in phage–bacteria population dynamics are not yet fully understood for *A. salmonicida* phage therapy. While phage specificity may reduce broad disruption of microbial communities compared with antibiotics, some reports cannot consider ecological effects insignificant without proper evaluation [218,219]. Future field studies should therefore monitor phage persistence, target and non-target bacterial populations, and changes in microbial community structure over time.

Finally, the PTRF correlation analysis suggests that stability studies are not sufficiently coupled with efficacy and translational studies, indicating a structural disconnection and a lack of systematic incorporation of stability. Addressing this gap will require integrated experiments, including determining phage stability and assessing phage antibacterial efficacy under conditions that represent real aquaculture environments.

B. Delivery methods for phage therapy of *A. salmonicida*

While the delivery approach is an important determinant of translational success‚ it has not yet been fully explored across the evidence base. The SLR found that most studies focused on relatively simple delivery approaches‚ such as direct exposure‚ which do not reflect aquaculture systems. As a result‚ the delivery and formulation domain showed relatively low translational evidence coverage in the PTRF assessment (Table 2).

Immersion therapy is one of the most common methods of phage therapy for fish [220,221]. Phage suspensions are applied directly in the water to be absorbed by free-floating bacteria or to the bacteria that are bound to the fish‚ such as the bacteria on gills or skin lesions [222]. Immersion treatments may be most effective in controlling early infections or in reducing bacterial loads in aquaculture facilities. In immersion experiments‚ AS-A considerably reduced *A*. *salmonicida* populations and improved the survival of fish infected with *A*. *salmonicida* [25,130].

Oral delivery via phage-supplemented pellet feed is another possible route of administration for bacteriophages and may be preferable in certain situations [223,224]. In this case‚ bacteriophages are mixed into pellet feed and consumed by fish and could treat gastrointestinal and systemic infections. Oral delivery is desirable for large aquaculture operations‚ enabling large populations of fish to be treated in a more humane‚ non-intrusive manner‚ but phages administered orally may become inactivated by digestive enzymes and low pH conditions within the gastrointestinal tract [225]. As a means of improving phage stability and controlling their release in target organisms‚ microencapsulation and other protective delivery systems have been explored [226,227].

C. Advances in *A. salmonicida* phage formulation and encapsulation

The use of formulation and encapsulation products is potentially an important approach for addressing the issues of environmental instability and poor delivery performance. However‚ there is a lack of formulation and encapsulation products developed for *A*. *salmonicida*‚ which may explain the low PTRF values for stability and delivery.

To maximize the efficacy of phage therapy against *A. salmonicida* infections‚ researchers have attempted to improve the stability‚ delivery‚ and persistence of phages using formulation and encapsulation. Environmental stresses in aquaculture systems can negatively affect the viability and efficacy of phages [228]. Temperature fluctuations‚ ultraviolet (UV) irradiation‚ and enzymatic degradation can inactivate phages. For this reason‚ there is important interest in protective delivery systems [18,203,211,212].

Microencapsulation is one of the most studied methods of phage protection‚ particularly using biopolymers such as alginate [229]. Alginate microcapsules could protect phages during storage and delivery‚ particularly in the case of oral administration‚ where phages must survive feed processing and passage through the gastrointestinal tract [230,231]. Moreover, hydrogel-based formulations could also help to protect phages and improve their persistence in water [226]. Other delivery methods such as liposomes or nanoparticles could offer advantages‚ possibly including increased phage stability and improved targeting or delivery to specific bacterial cells [232,233].

Despite the potential of these approaches‚ application of these technologies to *A*. *salmonicida* is limited and has to date not been coupled with formulation development and in vivo validation. Since there is a gap between experimental research and the development of market solutions‚ it is suggested that future research should focus on aquaculture-specific formulations and systematic evaluation of efficacy in the field [30,234].

In conclusion, there is a promising direction for the combination of phage cocktails and optimized formulation techniques to enhance treatment effect. Research on the scalability of encapsulation techniques, performance testing under field conditions, and formulation targeting *A. salmonicida*-specific infections would make significant progress for aquaculture.

#### 3.6.6. Limitations of Phage Therapy Against *A. salmonicida*

Although these promising results exist, limitations hinder bacteriophage therapy against *A. salmonicida*. The SLR and PTRF analysis indicate a strong focus on in vitro testing, with few in vivo studies and no field trials. As a result, the effectiveness of this therapy in real-world aquaculture settings remains poorly evaluated.

A. Phage resistance and environmental instability

Limitations of phages include their narrow host range‚ making them less effective against heterogeneous bacterial populations [141,235]. Phage resistance mechanisms, such as receptor modification and prophage-mediated resistance, remain common in bacterial populations and limit the efficacy of phage therapy [236,237]. As a result, phage cocktails may provide a solution but have not been researched as extensively.

Phage efficacy may be limited by environmental instability. Phages are rapidly inactivated by ultraviolet light‚ temperature fluctuations, and variations in local water chemistries [120,130,238]. These variables are rarely measured alongside phage treatment outcome. Likewise‚ delivery methods are generally immature‚ with most studies delivering pharmaceuticals in simple formats that do not integrate with aquaculture operations.

B. Genomic biosafety and horizontal gene transfer

Genomic safety is a fundamental requirement for the translational development of therapeutic *A. salmonicida* bacteriophages [239]. Therapeutic candidates should undergo whole-genome sequencing and bioinformatic screening for genes associated with lysogeny, known virulence determinants, antimicrobial-resistance genes, and other undesirable genetic elements [240,241]. Caution is required for temperate or potentially lysogenic phages because prophage integration and transduction may facilitate horizontal transfer of bacterial or phage-associated genetic material [121,182,242].

Strictly lytic *A. salmonicida* phages are therefore generally preferred for therapeutic development, for instance, TSW001 [117], T7-Ah [125], and ASP-1 [128]. However, genomic screening should not be limited to identifying a single lysogeny marker or known resistance gene. Comprehensive genome annotation and continued evaluation of newly characterized phage genes are necessary because the functional roles of some phage proteins may remain uncertain [243,244,245]. Genomic safety assessment should consequently be considered a prerequisite for candidate selection rather than an optional characterization step.

C. Regulatory and quality-control considerations

Few articles consider regulatory and scalability issues (i.e., product formulation and approval), indicating a large gap between the domains of experimental studies and commercial application (Table 2). A key gap in the development of *A*. *salmonicida* phage therapy is the approach to regulatory translation‚ as antibacterial activity alone is not sufficient. Regulatory considerations will require the proof of product identity‚ genome sequencing‚ safety‚ method of production‚ purity and potency‚ microbial limits‚ stability‚ formulation‚ and consistency of manufacture to be established for *A*. *salmonicida* phage products.

Another potential obstacle to regulatory approval of bacteriophage products can result from different regulatory routes for bacteriophage products in different jurisdictions‚ which can depend on the species of animal‚ intended use‚ route of administration‚ formulation‚ or use of an individual or combination of bacteriophages [246,247,248]. Thus‚ regulatory considerations should be taken into consideration during phage-development pipelines rather than at the time of showing efficacy.

D. Manufacturing, standardization, and scale-up

Laboratory-scale propagation of bacteriophages does not necessarily demonstrate feasibility for industrial production. Translation to practical aquaculture applications requires reproducible upstream production, appropriate bacterial production hosts, controlled phage amplification, downstream purification, removal or control of bacterial debris and other process-related contaminants, and reliable determination of phage potency [249,250]. Phage preparations should also demonstrate batch-to-batch consistency, defined quality specifications, acceptable storage stability, and preservation of infectivity during transportation and application [206,251]. These requirements become more complex for multi-phage cocktails because individual phage components may differ in replication kinetics, stability, purification behaviors, and storage requirements [252].

Accordingly, manufacturing feasibility should be evaluated alongside biological efficacy rather than only after laboratory development; scale-up studies and pilot production under conditions relevant to commercial aquaculture are important next steps for evaluating the reproducibility, quality, stability, and practical feasibility of phage-based interventions [253].

E. Economic feasibility of bacteriophage production

Economic feasibility is another important but poorly characterized component of phage-therapy translation. Relevant considerations include the cost of phage propagation and purification, formulation, quality control, storage, transportation, administration, and treatment frequency. For aquaculture applications, economic evaluation should additionally consider the scale of production, labor requirements, infrastructure, treatment losses, and potential savings associated with reduced antibiotic use or mortality [254,255].

The existing literature on *A. salmonicida* lacks enough standardized quantitative data to enable a reliable economic comparison between phage-based and traditional disease-control methods. As a result, this review did not include a specific numerical PTRF score for economic feasibility. Instead, it highlights this as a key evidence gap that should be addressed in future farm-scale and pilot-production research.

In summary‚ while phage therapy has demonstrated proof of concept‚ biological‚ environmental‚ and methodological limitations have precluded its application in aquaculture thus far. Future research needs to take biological‚ environmental‚ and methodological factors into account in an integrated‚ standardized‚ and application-oriented manner to strengthen translational evidence and guide the development of practical, cost-effective, and sustainable phage therapy strategies.

#### 3.6.7. Limitations, Future Perspectives, and Research Roadmap

A strategic research roadmap is necessary to address current bottlenecks and bridge the gap from experimental bacteriophage therapy applications to practical “next generation” aquaculture innovations. Future directions are in phage library design‚ refinement of cocktail design‚ and potentially in combination with other approaches for disease control (such as vaccination‚ probiotics‚ and biosecurity); based on the rapid evolution of phage genomics and biotechnology‚ phage cocktails could be a sustainable alternative to antibiotics in the treatment of *A. salmonicida* infections in aquaculture.

Engineered or adaptively evolved phages represent a potential future strategy for expanding host range, overcoming bacterial resistance, or improving therapeutic performance [256,257,258]. However, such approaches introduce additional biosafety considerations. Genetic modification or laboratory adaptation may generate unintended phenotypic changes, alter host specificity, affect replication dynamics, or introduce uncertainties regarding genetic stability [259,260,261]. Comprehensive genomic characterization is therefore required to confirm the absence of undesirable genes or unintended genomic alterations.

Horizontal gene transfer also requires careful consideration, particularly when engineered or naturally selected phages are intended for release into open or semi-open aquatic systems [242,262]. Therapeutic candidates should preferentially exhibit a strictly lytic lifestyle, and genomic analyses should evaluate lysogeny-associated genes, virulence determinants, antimicrobial-resistance genes, and other potentially mobilizable genetic elements [263,264].

Furthermore, engineered phages may be subject to additional regulatory requirements associated with genetically modified biological products and, depending on the intended application, environmental release [246,265]. Therefore, engineering strategies should be accompanied by rigorous genomic, phenotypic, ecological, and regulatory assessment before field application.

The framework below summarises key challenges and corresponding future directions that should guide subsequent research and solutions in this field (Table 6).

This roadmap highlights that future progress in phage therapy will depend on interdisciplinary integration of microbiology, biotechnology, and aquaculture engineering, particularly in improving phage stability, delivery efficiency, and large-scale validation under commercial farming conditions.

### 3.7. Limitations of This Systematic Review

The main limitation of this systematic review was the high heterogeneity among the studies in terms of *A*. *salmonicida* strain or subspecies‚ bacteriophage isolate or biological characteristics‚ host fish species‚ infection models‚ challenge dose‚ MOI or phage concentrations‚ routes of administration‚ timing of treatment‚ environmental conditions‚ formulation and measured outputs. Methods for measuring bacterial reduction‚ phage activity‚ survival‚ and resistance were also not standardized.

This heterogeneity substantially limits direct comparison of therapeutic outcomes and prevents meaningful pooling of effect estimates across studies. Consequently, a narrative synthesis was considered more appropriate than quantitative meta-analysis.

The available literature may also be affected by publication bias because successful phage isolation, strong antibacterial activity, and positive therapeutic outcomes may be more likely to be reported than unsuccessful or ambiguous findings. In addition, studies reported phage preparation, genomic safety, resistance development, stability, and in vivo treatment parameters inconsistently.

Finally, the PTRF itself remains exploratory and has not undergone independent external validation. Therefore, interpret its classifications as structured evidence mapping rather than definitive measurements of translational evidence coverage.

## 4. Conclusions

This systematic review indicates that bacteriophage therapy has substantial biological potential against *A. salmonicida*, particularly based on repeated in vitro evidence of bacterial inhibition and the limited number of controlled in vivo studies reporting improved disease outcomes. Despite current evidence supporting the biological promise of bacteriophage therapy against *A. salmonicida,* it does not provide translational evidence coverage for widespread industrial application. Most available studies remain laboratory-based, and routine aquaculture implementation requires well-controlled field trials, standardized treatment protocols, scalable manufacturing, environmental assessment, and regulatory evaluation.

The PTRF analysis indicates that the strongest evidence currently concerns biological efficacy, whereas major gaps remain in broad host coverage, resistance robustness, environmental persistence, delivery and formulation, manufacturing, regulatory requirements, economic feasibility, and field-scale validation. Importantly, PTRF scores should be interpreted as evidence-coverage indicators rather than as definitive measures of commercial or regulatory readiness.

Therefore, bacteriophage therapy should currently be regarded as a promising but still experimentally developing approach for *A. salmonicida* disease management. Progress toward practical aquaculture application will require standardized multi-strain testing, genomic safety assessment, resistance monitoring, formulation and delivery optimization, scalable production, environmental evaluation, economic assessment, and well-designed field trials.

## Figures and Tables

**Table 1:** Summary of experimental studies on bacteriophage therapy targeting *A. salmonicida* in aquaculture systems.

Author	Host Fish	Phage Type	Study Type	Key Findings
Yan, Guo [117]	Large yellow croaker	Lytic	In vitro	Stable across wide conditions; strong bacterial reduction; inhibits biofilm formation
Jiang, Liu [118]	Not specified	Lytic	In vitro	High burst size; strong antibacterial activity; stable under varying conditions
Li, Fang [9]	Not evaluated	Lytic (ASG01)	In vitro	Identified a novel virulent phage with high stability and a potent endolysin (Cwh) showing strong antibacterial activity against *A. salmonicida* and other fish pathogens.
Svanberga, Avsejenko [119]	Salmonids	Lytic (podophage)	In vitro	Rapid adsorption; strong lysis; narrow host range
Xu, Ding [120]	Not specified	Lytic	Stability + in vivo	Optimal storage at 4 °C; improved stability with DMSO and lyophilization
Hosseini, Paquet [121]	Salmonids	Lytic (cocktail)	In vitro	Effective cocktail: resistance linked to prophage presence
Leduc, Paquet [122]	Brook trout	Temperate myophage (LPM4/Prophage 3)	Genomic + in vitro	LPM4 was identified as the active form of Prophage 3, showing broad infectivity but limited therapeutic suitability due to lysogenization and prophage-mediated resistance.
Morozova, Babkin [123]	Not specified	Jumbo lytic (phiKZ-like)	Genomic + in vitro	Large genome (~243 kb); slow lytic cycle; no virulence genes; narrow host range
Nikapitiya, Senevirathne [124]	Crucian carp (isolation source)	Lytic (Myoviridae, Tequatrovirus-like)	In vitro	Large genome (~218 kb); infects both *A. hydrophila* and *A. salmonicida*; no harmful genes
Leduc, Paquet [125]	Not specified	Lytic (podophage T7-Ah)	Genomic + in vitro	Broad host range; safe genome; efficient replication
Xu, Jin [26]	Turbot (*Scophthalmus maximus*)	Lytic	In vivo + in vitro	Reduced mortality; lower bacterial load; safe profile
Hosseini, Paquet [126]	Brook trout	Virulent/lytic phages; 5-phage panel	In vitro	4-phage cocktail (HER98 + HER110 + SW69-9 + Riv-10) at MOI 1 was most effective; Prophage 3 reduced cocktail efficacy
Poblete-Morales, Irgang [127]	Not specified	Myoviridae, Caudovirales	In vitro	Stable across pH and temperature ranges
Nikapitiya, Dananjaya [128]	Goldfish (*Carassius auratus*)	Lytic (Myoviridae)	In vitro	MOI-dependent killing; stable; moderate burst size
Paquet, Vincent [129]	Not specified	Broad host-range phage	In vitro	LPS receptor identified; resistance via A-layer
Duarte, Pereira [130]	Not specified	Lytic (Myoviridae; AS-A, AS-D, AS-E)	In vitro + environmental stability	Phage cocktails enhance bacterial reduction and reduce resistance; stability affected by UV and temperature
Yang, Yuan [131]	Not specified	Temperate + lytic potential	Genomic + in vitro	Dual life cycle; multi-strain infectivity
Chen, Yuan [28]	Not specified	Lytic cocktail	In vitro	Strong synergistic antibacterial effect
Moreirinha, Osório [132]	Not specified	Lytic (AS-A)	In vitro	Protein expression changes: resistance mechanisms identified
Vincent, Paquet [133]	Not specified	Mixed (lytic + temperate)	Genomic + in vitro	High diversity: host specificity varies
Silva, Moreirinha [25]	Senegalese sole	Lytic	In vivo	Phage treatment reduced mortality; effective control of infection
Kim, Choresca [134]	Rainbow trout (*Oncorhynchus mykiss*)	Lytic (PAS-1)	In vivo	Increased survival; strong bacteriolysis; phage cleared within 200 h; immune response observed

**Table 2:** Integrated evaluation of included studies combining methodological quality assessment and PTRF scoring.

Author	Evidence Level	E	H	S	D	T	Total
Yan, Guo [117]	Limited	1	2	2	1	1	7
Jiang, Liu [118]	Moderate	1	0	2	0	1	4
Li, Fang [9]	Limited	1	1	2	0	1	5
Svanberga, Avsejenko [119]	Moderate	1	2	2	0	1	6
Xu, Ding [120]	Limited	1	0	3	0	2	6
Hosseini, Paquet [121]	Moderate	1	3	0	0	0	4
Leduc, Paquet [122]	Limited	1	3	0	0	0	4
Morozova, Babkin [123]	Limited	1	0	0	0	0	1
Nikapitiya, Senevirathne [124]	Limited	1	1	0	0	0	2
Leduc, Paquet [125]	Moderate	1	2	0	0	0	3
Xu, Jin [26]	Strong	3	2	2	2	2	11
Hosseini, Paquet [126]	Moderate	1	3	0	0	1	5
Poblete-Morales, Irgang [127]	Limited	1	2	2	0	1	5
Nikapitiya, Dananjaya [128]	Moderate	1	1	3	0	1	6
Paquet, Vincent [129]	Moderate	1	1	0	0	1	3
Duarte, Pereira [130]	Moderate	1	2	3	1	2	9
Yang, Yuan [131]	Limited	1	0	0	0	0	1
Chen, Yuan [28]	Moderate	1	3	2	0	1	7
Moreirinha, Osório [132]	Limited	1	2	0	0	0	3
Vincent, Paquet [133]	Moderate	1	3	0	0	0	4
Silva, Moreirinha [25]	Strong	3	2	0	2	2	9
Kim, Choresca [134]	Strong	3	0	0	2	1	6
**Mean**		1.27	1.59	1.05	0.36	0.82	

**PTRF pillars:** E = Efficacy; H = Host coverage and resistance robustness; S = Environmental stability; D = Delivery and formulation. T = Translational feasibility; **Readiness classification:** Low (0–5), Moderate (6–10), High (11–15) translational evidence coverage; **Evidence Level:** Limited = primarily in vitro or descriptive studies; Moderate = well-characterized studies with partial translational relevance; Strong = studies including in vivo validation and therapeutically relevant outcomes.

**Table 3:** Comparative assessment of experimental design, evidence strength, study limitations, and PTRF-based translational evidence coverage across the included studies.

Author	Experimental Design	Infection Model	Phage Type	Evidence Strength	Main Limitations	PTRF Score	Translational Evidence Coverage
Yan, Guo [117]	In vitro	Large yellow croaker	Lytic	Limited	In vitro only, no in vivo validation	7	Moderate
Jiang, Liu [118]	In vitro	Not specified	Lytic	Moderate	In vitro/genomic study; no therapeutic validation	4	Low
Li, Fang [9]	In vitro	Not evaluated	Lytic (ASG01)	Limited	Narrow host range; no in vivo validation	5	Low
Svanberga, Avsejenko [119]	In vitro	Salmonids	Lytic (podophage)	Moderate	Narrow host range; no in vivo validation	6	Moderate
Xu, Ding [120]	Stability + in vivo	Not specified	Lytic	Limited	Mainly stability-focused; limited therapeutic evidence	6	Moderate
Hosseini, Paquet [121]	In vitro	Salmonids	Lytic (cocktail)	Moderate	In vitro only; resistance linked to prophage	4	Low
Leduc, Paquet [122]	Genomic + in vitro	Not specified	Temperate myophage (LPM4/Prophage 3)	Limited	In vitro only, no in vivo validation	4	Low
Morozova, Babkin [123]	Genomic + in vitro	Not specified	Jumbo lytic (phiKZ-like)	Limited	Narrow host range; slow lytic cycle	1	Low
Nikapitiya, Senevirathne [124]	In vitro	Crucian carp (isolation source)	Lytic (Myoviridae, Tequatrovirus-like)	Limited	In vitro/genomic only; no therapeutic validation	2	Low
Leduc, Paquet [125]	Genomic + in vitro	Not specified	Lytic (podophage T7-Ah)	Moderate	Limited strain coverage; no in vivo validation	3	Low
Xu, Jin [26]	In vivo + in vitro	Turbot (*Scophthalmus maximus*)	Lytic	Strong	Experimental in vivo model; no field validation	11	Strong
Hosseini, Paquet [126]	In vitro	Brook trout	Virulent/lytic phages; 5-phage panel	Moderate	In vitro only, no in vivo validation	5	Low
Poblete-Morales, Irgang [127]	In vitro	Not specified	Myoviridae, Caudovirales	Limited	In vitro only, no in vivo validation	5	Low
Nikapitiya, Dananjaya [128]	In vitro	Goldfish (*Carassius auratus*)	Lytic (Myoviridae)	Moderate	In vitro only, no therapeutic validation	6	Moderate
Paquet, Vincent [129]	In vitro	Not specified	Broad host-range phage	Moderate	Mechanistic study; limited therapeutic evidence	3	Low
Duarte, Pereira [130]	In vitro + environmental stability	Not specified	Lytic (Myoviridae; AS-A, AS-D, AS-E)	Moderate	No in vivo validation or delivery assessment	9	Moderate
Yang, Yuan [131]	Genomic + in vitro	Not specified	Temperate + lytic potential	Limited	Genomic/in vitro evidence; limited therapeutic relevance	1	Low
Chen, Yuan [28]	In vitro	Not specified	Lytic cocktail	Moderate	In vitro cocktail study; no in vivo validation	7	Moderate
Moreirinha, Osório [132]	In vitro	Not specified	Lytic (AS-A)	Limited	Resistance-focused; limited therapeutic evidence	3	Low
Vincent, Paquet [133]	Genomic + in vitro	Not specified	Mixed (lytic + temperate)	Moderate	Host-specificity study; limited therapeutic validation	4	Low
Silva, Moreirinha [25]	In vivo	Senegalese sole	Lytic	Strong	Experimental in vivo model; no field validation	9	Moderate
Kim, Choresca [134]	In vivo	Rainbow trout (*Oncorhynchus mykiss*)	Lytic (PAS-1)	Strong	In vivo model; limited persistence and field validation	6	Moderate

**Table 5:** Summary of therapeutic outcomes and application strategies of bacteriophage therapy against *A. salmonicida*.

Study	Model	Application Method	Outcome Type	Key Findings	Implication for Therapy
Yan, Guo [117]	Experimental	Direct exposure	Bacterial reduction	~1–2 log reduction; biofilm inhibition	Demonstrates strong in vitro efficacy and anti-biofilm potential
Jiang, Liu [118]	In vitro + genomic	Direct exposure	Growth inhibition	High burst size; strong antibacterial activity	Promising candidate; requires in vivo validation
Li, Fang [9]	In vitro	Not applicable	Antibacterial activity	Stable virulent phage with active endolysin.	Potential antibacterial for *A. salmonicida*
Svanberga, Avsejenko [119]	In vitro + genomic	Direct exposure	Lysis kinetics	Rapid adsorption; narrow host range	Limited applicability; supports cocktail design
Xu, Ding [120]	Stability study + in vivo	Storage optimization	Stability	Improved stability via DMSO, lyophilization	Relevant for formulation development
Hosseini, Paquet [121]	In vitro	Phage cocktail	Growth inhibition	Strong suppression; resistance influenced by prophage	Highlights importance of cocktail design to overcome resistance
Leduc, Paquet [122]	In vitro	Not applicable	Host range, Genomic characterization	LPM4 is temperate; Prophage 3 can confer superinfection resistance	Not ideal for phage therapy; lytic phages are preferred
Morozova, Babkin [123]	Genomic + in vitro	Not applicable	Genomic characterization	Large genome; no virulence genes; slow cycle	Safe but uncertain therapeutic efficiency
Nikapitiya, Senevirathne [124]	Genomic + in vitro	Not applicable	Host range	Cross-infection of Aeromonas spp.	Potential for broader-spectrum therapy
Leduc, Paquet [125]	Genomic + in vitro	Direct exposure	Bacterial reduction	Broad host range; efficient replication	Promising candidate; lacks in vivo validation
Xu, Jin [26]	In vivo	Immersion	Survival, bacterial load	Increased survival; significant reduction in bacterial load	Provides controlled in vivo proof of concept; further validation under field-scale aquaculture conditions is required
Hosseini, Paquet [126]	In vitro	Phage cocktail	Bacterial growth inhibition	A four-phage cocktail was most effective, but Prophage 3 reduced phage sensitivity	Prophage 3 can compromise cocktail efficacy; new virulent phages and fish trials are needed
Poblete-Morales, Irgang [127]	In vitro	Environmental exposure	Stability	Stable across pH and temperature	Supports environmental application feasibility
Nikapitiya, Dananjaya [128]	In vitro	MOI variation	Killing efficiency	MOI-dependent bacterial reduction; stable under varied conditions	Emphasizes need for dosage optimization
Paquet, Vincent [129]	In vitro	Not applicable	Receptor interaction	LPS receptor identified; resistance via A-layer	Critical insight for resistance-aware design
Duarte, Pereira [130]	In vitro + stability	Immersion	Bacterial reduction	Cocktail improves efficacy; UV reduces viability	Supports cocktail use; highlights environmental limits
Yang, Yuan [131]	Genomic + in vitro	Not applicable	Lifecycle analysis	Dual lytic/lysogenic potential	Risk for therapy; careful selection needed
Chen, Yuan [28]	In vitro	Phage cocktail	Synergistic effect	Enhanced antibacterial activity compared to single phage	Supports use of multi-phage formulations
Moreirinha, Osório [132]	In vitro	Not applicable	Cellular response	Protein expression changes; resistance adaptation	Reveals resistance mechanisms
Vincent, Paquet [133]	Genomic + in vitro	Not applicable	Host specificity	High diversity; variable host range	Reinforces need for cocktails
Silva, Moreirinha [25]	In vivo	Immersion	Survival	Reduced mortality in fish	Provides in vivo evidence of therapeutic activity under controlled experimental conditions
Kim, Choresca [134]	In vivo	Injection/exposure	Survival, immune response	Increased survival; immune activation; phage clearance	Early proof of in vivo efficacy

**Table 6:** Research roadmap for advancing bacteriophage therapy against *A. salmonicida* in aquaculture.

Area	Current Limitation	Future Direction
Host range specificity	Narrow and strain-dependent host range	Rational cocktail design and, where justified, engineered or adaptively evolved phages accompanied by genomic, biosafety, ecological, and regulatory assessment
Resistance	Rapid evolution	Multi-phage cocktails
Stability	Environmental sensitivity	Encapsulation technologies
Delivery	Inefficient targeting	Oral + nano-enabled delivery systems
Field validation	Limited trials	Large-scale field validation in aquaculture
Regulation	Unclear frameworks	Policy development

## Data Availability

No new data were created or analyzed in this study. Data sharing is not applicable to this article.

## Supplementary Materials

The following supporting information can be downloaded at: https://www.mdpi.com/article/10.3390/microorganisms14102274/s1, Table S1: List of Studies Excluded During Full-Text Screening and Reasons for Exclusion. Table S2: Methodological evidence-strength assessment. Table S3: Scoring Criteria for the Phage Translational Readiness Framework (PTRF).

## Abbreviations

The following abbreviations are used in this manuscript:

AMR	Antimicrobial Resistance
LPS	Lipopolysaccharide
MCPs	Multiple-country Publications
MOI	Multiplicity of Infection
PTRF	Phage Therapy Readiness Framework
RBPs	Receptor-binding Proteins
SCPs	Single-country Publications
SLR	Systematic Literature Review
WoS	Web of Science
