# Supplementary material for: Bacteriophage Therapy for Aeromonas salmonicida: A Systematic Evaluation of Translational Readiness in Aquaculture
Source: Microorganisms. 2026 Oct 6;14(10):2274. doi: 10.3390/microorganisms14102274 (PMC13707803; doi:10.3390/microorganisms14102274)
Supplement: Supplementary file 1 [file microorganisms-14-02274-s001.zip › Table S1. List of Studies Excluded During Full-Text Screening and Reasons for Exclusion.pdf]

Table S1. List of Studies Excluded During Full-Text Screening and Reasons for Exclusion

S1.1. Studies Excluded for Topic Irrelevance

No	Reference	Year	Screening stage	Reason
1	Castillo, Cristiansen [33]	2015	Title/Abstract	Not *A. salmonicida*
2	Hai [34]	2015	Title/Abstract	Not primary research
3	Ji, Yu [35]	2015	Title/Abstract	Not *A. salmonicida*
4	Hoque, Bin Naser [36]	2016	Title/Abstract	Not *A. salmonicida*
5	Sasikala and Srinivasan [37]	2016	Title/Abstract	Not *A. salmonicida*
6	Cabello, Tomova [38]	2017	Title/Abstract	Not primary research
7	Hernández [39]	2017	Title/Abstract	Not *A. salmonicida*
8	Lal, Sano [40]	2017	Title/Abstract	Not *A. salmonicida*
9	Wang, Barton [42]	2017	Title/Abstract	Not *A. salmonicida*
10	Uhrynowski, Decewicz [41]	2017	Title/Abstract	Not bacteriophage-based
11	Kokkari, Sarropoulou [43]	2018	Title/Abstract	Not *A. salmonicida*
12	Le, Nguyen [44]	2018	Title/Abstract	Not *A. salmonicida*
13	Luo, Liao [45]	2018	Title/Abstract	Not *A. salmonicida*
14	Bai, Cheng [46]	2019	Title/Abstract	Not *A. salmonicida*
15	Cao, Li [48]	2019	Title/Abstract	Not *A. salmonicida*
16	Castillo, Rorbo [49]	2019	Title/Abstract	Not *A. salmonicida*
17	Costa, Pereira [50]	2019	Title/Abstract	Not *A. salmonicida*
18	He, Huang [51]	2019	Title/Abstract	Not *A. salmonicida*
19	Hoang, Xuan [52]	2019	Title/Abstract	Not *A. salmonicida*
20	Pinheiro, Pereira [53]	2019	Title/Abstract	Not *A. salmonicida*
21	Bhagawati, Terhorst [47]	2019	Title/Abstract	Out of topic
22	Akmal, Rahimi-Midani [54]	2020	Title/Abstract	Not *A. salmonicida*
23	Cao, Li [55]	2020	Title/Abstract	Not *A. salmonicida*
24	Hammer-Dedet, Jumas-Bilak [56]	2020	Title/Abstract	Out of topic
25	Lim, Lee [57]	2020	Title/Abstract	Not *A. salmonicida*
26	Liu, Gao [58]	2020	Title/Abstract	Not *A. salmonicida*
27	Pinheiro, Pereira [59]	2020	Title/Abstract	Not *A. salmonicida*
28	Żaczek, Weber-Dabrowską [60]	2020	Title/Abstract	Not primary research
29	Duarte, Pereira [61]	2021	Title/Abstract	Not *A. salmonicida*
30	Islam, Yang [62]	2021	Title/Abstract	Not *A. salmonicida*
31	Kunttu, Runtuvuori-Salmela [63]	2021	Title/Abstract	Not *A. salmonicida*
32	Li, Tian [64]	2021	Title/Abstract	Not *A. salmonicida*
33	Lindivat, Bratbak [65]	2021	Title/Abstract	Out of topic
34	Ramos-Vivas, Superio [68]	2021	Title/Abstract	Not primary research
35	Thomas [69]	2021	Title/Abstract	Out of topic
36	Wang, Yu [70]	2021	Title/Abstract	Not *A. salmonicida*
37	Xu, Zhang [71]	2021	Title/Abstract	Not *A. salmonicida*
38	Zhang, Xu [72]	2021	Title/Abstract	Not *A. salmonicida*
39	Nhinh, Le [67]	2021	Title/Abstract	Out of topic
40	Marcoux, Vincent [66]	2021	Title/Abstract	Not bacteriophage-based
41	Amgarten, Iha [73]	2022	Title/Abstract	Not primary research
42	Droubogiannis and Katharios [74]	2022	Title/Abstract	Not *A. salmonicida*
43	Droubogiannis, Pavlidi [75]	2022	Title/Abstract	Not *A. salmonicida*
44	Feng, Jia [76]	2022	Title/Abstract	Not *A. salmonicida*
45	Janelidze, Jaiani [77]	2022	Title/Abstract	Not *A. salmonicida*

46	Jose Priya and Kappalli [78]	2022	Title/Abstract	Out of topic
47	Morozova, Kozlova [79]	2022	Title/Abstract	Not *A. salmonicida*
48	Pan, Li [80]	2022	Title/Abstract	Not *A. salmonicida*
49	Pereira, Duarte [30]	2022	Title/Abstract	Not primary research
50	Ren, Wang [81]	2022	Title/Abstract	Not *A. salmonicida*
51	Ridzuan, Abdullah [82]	2022	Title/Abstract	Out of topic
52	Skurnik [83]	2022	Title/Abstract	Not primary research
53	Sukjoi, Buddhasiri [84]	2022	Title/Abstract	Not *A. salmonicida*
54	Ture, Cebeci [85]	2022	Title/Abstract	Not *A. salmonicida*
55	Xu, Shao [86]	2022	Title/Abstract	Not *A. salmonicida*
56	Yu, Zhang [24]	2022	Title/Abstract	Not *A. salmonicida*
57	Yu, Feng [87]	2022	Title/Abstract	Not *A. salmonicida*
58	Droubogiannis, Pavlidi [88]	2023	Title/Abstract	Not *A. salmonicida*
59	Hou, Wu [89]	2023	Title/Abstract	Not *A. salmonicida*
60	Li, Zhang [90]	2023	Title/Abstract	Not *A. salmonicida*
61	Nokhwal, Anand [91]	2023	Title/Abstract	Not primary research
62	Rai, Tyagi [92]	2023	Title/Abstract	Not *A. salmonicida*
63	Ye, Tong [94]	2023	Title/Abstract	Not *A. salmonicida*
64	Yang, Sepúlveda [93]	2023	Title/Abstract	Not bacteriophage-based
65	Vaibarova, Kralova [103]	2024	Title/Abstract	Out of topic
66	Rai, Kaur [99]	2024	Title/Abstract	Out of topic
67	Hsu, Shih [95]	2024	Title/Abstract	Not *A. salmonicida*
68	Liu, Wu [96]	2024	Title/Abstract	Not *A. salmonicida*
69	Lyu, Xiong [97]	2024	Title/Abstract	Not *A. salmonicida*
70	Nosalj, Hrabovsky [98]	2024	Title/Abstract	Out of topic
71	Sankappa, Kallappa [100]	2024	Title/Abstract	Not *A. salmonicida*
72	Sawaengwong, Sunthornthummas [101]	2024	Title/Abstract	Not *A. salmonicida*
73	Ture, Aygur [102]	2024	Title/Abstract	Not *A. salmonicida*
74	Yaşa, Evran [104]	2024	Title/Abstract	Not *A. salmonicida*
75	Zhang, Li [105]	2024	Title/Abstract	Not *A. salmonicida*
76	Hien, Lanh [106]	2025	Title/Abstract	Not *A. salmonicida*
77	Li, Yue [107]	2025	Title/Abstract	Not *A. salmonicida*
78	Nakamura, Zhang [108]	2025	Title/Abstract	Out of topic
79	Parker, Marten [109]	2025	Title/Abstract	Not *A. salmonicida*
80	Ramírez and Romero [110]	2025	Title/Abstract	Not primary research
81	Wang, Li [111]	2025	Title/Abstract	Not bacteriophage-based
82	Wang, Li [112]	2025	Title/Abstract	Not *A. salmonicida*

S1.2. Studies Excluded for Not Retrieved

No	Reference	Year	Screening stage	Reason
1	Bakiyev, Smekenov [113]	2023	Title/Abstract	Not retrieved

S1.3. Review articles on *Aeromonas salmonicida* excluded from qualitative synthesis

No	Reference	Year	Screening stage	Reason
1	Choudhury, Nagaraju [114]	2015	Full-text	Review article
2	Park, Han [115]	2020	Full-text	Review article
3	Qin, Li [116]	2025	Full-text	Review article
