# Supplementary material for: Bacteriophage Therapy for Aeromonas salmonicida: A Systematic Evaluation of Translational Readiness in Aquaculture
Source: Microorganisms. 2026 Oct 6;14(10):2274. doi: 10.3390/microorganisms14102274 (PMC13707803; doi:10.3390/microorganisms14102274)
Supplement: Supplementary file 1 [file microorganisms-14-02274-s001.zip › Table S2. Methodological evidence-strength assessment.pdf]

Table S2: Methodological evidence-strength assessment

Study	Experimental design	Methodological transparency & reproducibility	Outcome reporting	Translational relevance	Evidence strength
Yan, Guo [117]	*In vitro*	MOI, experimental conditions and procedures reported	Bacterial reduction and phage activity reported	Aquaculture-relevant fish matrix; no live-fish infection model	Limited
Jiang, Liu [118]	*In vitro*	MOI, growth conditions, stability and experimental procedures reported	Antibacterial activity, latent period, burst size and stability reported	*A. salmonicida* testing; aquaculture relevance, but no *in vivo* validation	Moderate
Li, Fang [9]	*In vitro*	Phage isolation, stability conditions, lytic assays and genomic methods reported	Lytic activity, host range, stability and Cwh activity reported	*A. salmonicida* and fish-pathogenic bacteria tested; no *in vivo* validation	Limited
Svanberga, Avsejenko [119]	*In vitro*	MOI, adsorption, growth parameters, stability and genomic methods reported	Lytic activity, host range, burst size and stability reported	Aquaculture-relevant strain panel; no *in vivo* validation and narrow host range	Moderate
Xu, Ding [120]	Stability + *in vivo*	Storage temperature, cryoprotectants, lyophilization conditions, titers, and shelf-life modelling clearly reported	Phage viability, storage stability, lyophilization performance, and predicted shelf life reported	Relevant to phage formulation and storage, but no *in vivo* therapeutic validation	Limited
Hosseini, Paquet [121]	*In vitro*	Host range, EOP, infection conditions and genomic analyses reported	Lytic activity and host-range outcomes clearly reported	Extensive multi-strain testing (62 strains) and direct relevance to *A. salmonicida* phage resistance; no *in vivo* validation	Moderate
Leduc, Paquet [122]	Genomic + *in vitro*	Phage infection, adsorption, lyonization and genomic analyses reported	Host specificity, adsorption, infection and prophage dynamics reported	Relevant to *A. salmonicida* and phage-resistance mechanisms, but no *in vivo* validation	Limited
Morozova, Babkin [123]	Genomic + *in vitro*	Host-range assays, growth characteristics, stability and genomic methods reported	Lytic activity, host range, growth parameters and genomic characteristics reported	*A. salmonicida* testing, but narrow host range and no *in vivo* validation	Limited

Nikapitiya, Senevirathne [124]	*In vitro*	Phage isolation and genome-analysis methods reported	Host range and genomic characteristics reported	*A. salmonicida* included, but limited application evidence and no *in vivo* validation	Limited
Leduc, Paquet [125]	Genomic + *in vitro*	Host range, adsorption, burst size/time, LPS analysis and genomic characterization reported	Lytic activity, host range, adsorption and phage characteristics reported	Testing across mesophilic and psychrophilic *A. salmonicida* strains; aquaculture relevance, but no *in vivo* validation	Moderate
Xu, Jin [26]	*In vivo* + *in vitro*	MOI, growth conditions, stability, dosing and experimental procedures reported	Mortality, bacterial burden and inflammatory responses reported	Aquaculture-relevant turbot model and therapeutic phage application	Strong
Hosseini, Paquet [126]	*In vitro*	Phage combinations, MOI, strain backgrounds, experimental procedures and controls reported	Phage inhibition, local virulence indices and effects of Prophage 3 reported	Extensive testing of *A. salmonicida* subsp. *salmonicida* strains and direct relevance to phage-cocktail development, but no *in vivo* validation	Moderate
Poblete-Morales, Irgang [127]	*In vitro*	MOI, temperature, pH, salinity, storage conditions and experimental procedures reported	Bacterial reduction, biofilm inhibition and phage stability reported	Food-matrix application using large yellow croaker; no live-fish therapeutic validation	Limited
Nikapitiya, Dananjaya [128]	*In vitro*	MOI, adsorption, one-step growth, stability and experimental conditions reported	Lytic activity, burst size, adsorption and stability reported	*A. salmonicida* from goldfish; aquaculture-relevant conditions, but no *in vivo* validation	Moderate
Paquet, Vincent [129]	*In vitro*	Adsorption assays, mutant characterization and genomic analyses reported	Adsorption, LPS-associated phage interaction and phage-insensitive mutants reported	Direct relevance to *A. salmonicida* phage resistance, but no *in vivo* validation	Moderate
Duarte, Pereira [130]	*In vitro* + environmental stability	MOI, phage combinations, bacterial dynamics and environmental conditions reported	Bacterial reduction, phage resistance and phage stability reported	Strong aquaculture relevance and environmental testing; no *in vivo* validation in this study	Moderate

Yang, Yuan [131]	Genomic + *in vitro*	Genome sequencing, ORF annotation and phylogenetic analyses reported	Genome organization and predicted phage functions reported	*A. salmonicida* phage, but primarily genomic/descriptive; no *in vivo* validation	Limited
Chen, Yuan [28]	*In vitro*	Adsorption, MOI, growth conditions, host range and cocktail procedures reported	Lytic activity, bacterial inhibition, host range and phage synergy reported	38 bacterial strains tested; broad *A. salmonicida* relevance and cocktail development, but no *in vivo* validation	Moderate
Moreirinha, Osório [132]	*In vitro*	Phage treatment, resistance selection, EOP, spectroscopy and protein-analysis methods reported	Phage sensitivity, EOP and protein-expression changes reported	Direct relevance to phage resistance in *A. salmonicida*, but no *in vivo* validation	Limited
Vincent, Paquet [133]	Genomic + *in vitro*	Isolation, one-step growth, host-range testing and genome sequencing/analysis clearly reported	Host range, burst size, latent period and genomic diversity reported	65 *A. salmonicida* isolates and 12 phages tested; relevant to aquaculture and cocktail development, but no *in vivo* validation	Moderate
Silva, Moreirinha [25]	*In vivo*	Phage isolation/characterization, infection model, phage administration and experimental procedures reported	Bacterial control and infection-related outcomes in experimentally infected fish reported	Aquaculture-relevant fish model with direct therapeutic application of AS-A	Strong
Kim, Choresca [134]	*In vivo*	Phage administration, infection model, MOI and experimental procedures reported	Survival, mean time to death, phage clearance and serum neutralizing activity reported	Aquaculture-relevant rainbow trout infection model with direct therapeutic application of PAS-1	Strong
