# Supplementary material for: Bacteriophage Therapy for Aeromonas salmonicida: A Systematic Evaluation of Translational Readiness in Aquaculture
Source: Microorganisms. 2026 Oct 6;14(10):2274. doi: 10.3390/microorganisms14102274 (PMC13707803; doi:10.3390/microorganisms14102274)
Supplement: Supplementary file 1 [file microorganisms-14-02274-s001.zip › Table S3. Scoring Criteria for the Phage Translational Readiness Framework (PTRF).pdf]

Table S3. Scoring Criteria for the Phage Translational Readiness Framework (PTRF)

Domain	Score	Criteria
Efficacy	0	No antibacterial data
	1	*In vitro* evidence only (e.g., plaque assay, bacterial growth inhibition)
	2	Limited *in vivo* efficacy evidence or preliminary therapeutic outcome
	3	Robust *in vivo* therapeutic efficacy (e.g., improved survival, reduced bacterial load, replicated outcomes)
Host coverage	0	Single bacterial strain tested
	1	2–5 strains tested
	2	>5 strains tested or moderate host range
	3	Broad host range with resistance evaluation
Stability	0	Not assessed
	1	Single environmental condition tested (e.g., temperature only)
	2	Multiple environmental conditions tested (e.g., pH, temperature)
	3	Comprehensive stability including storage and/or formulation effects
Delivery	0	No delivery strategy described
	1	Basic/direct phage exposure to bacteria or host, without a defined delivery strategy
	2	Defined delivery method (e.g., immersion, oral, injection)
	3	Optimized delivery (e.g., encapsulation, controlled release, dosing strategy)
Translation	0	Laboratory study with no evidence addressing practical application
	1	Preliminary translational evidence (e.g., aquaculture-relevant conditions, formulation, storage, or basic safety assessment)
	2	Multiple translational requirements addressed (e.g., delivery/formulation, stability, safety, reproducibility, or realistic aquaculture conditions)
	3	Advanced translational evidence, such as field validation, scalability assessment, or regulatory considerations
